# *TRPA5* encodes a thermosensitive ankyrin ion channel receptor in a triatomine insect

**DOI:** 10.1016/j.isci.2024.109541

**Published:** 2024-03-20

**Authors:** Marjorie A. Liénard, David Baez-Nieto, Cheng-Chia Tsai, Wendy A. Valencia-Montoya, Balder Werin, Urban Johanson, Jean-Marc Lassance, Jen Q. Pan, Nanfang Yu, Naomi E. Pierce

**Affiliations:** 1 Department of Biology, Lund University, 22362 Lund, Sweden; 2 Department of Organismic and Evolutionary Biology and Museum of Comparative Zoology, Harvard University, Cambridge, MA 02138, USA; 3 Broad Institute, Cambridge, MA 02142, USA; 4 Stanley Center for Psychiatric Research, Broad Institute, Cambridge, MA 02142, USA; 5 Department of Applied Physics and Applied Mathematics, Columbia University, New York, NY 10027, USA; 6 Division of Biochemistry and Structural Biology, Department of Chemistry, Lund University, 22362 Lund, Sweden; 7 Laboratory of Evolutionary Neuroethology, GIGA Institute, University of Liège, 4000 Liège, Belgium

**Keywords:** Biological sciences, Entomology, Molecular biology

## Abstract

As ectotherms, insects need heat-sensitive receptors to monitor environmental temperatures and facilitate thermoregulation. We show that *TRPA5,* a class of ankyrin transient receptor potential (TRP) channels absent in dipteran genomes, may function as insect heat receptors. In the triatomine bug *Rhodnius prolixus* (order: Hemiptera), a vector of Chagas disease, the channel RpTRPA5B displays a uniquely high thermosensitivity, with biophysical determinants including a large channel activation enthalpy change (72 kcal/mol), a high temperature coefficient (Q_10_ = 25), and *in vitro* temperature-induced currents from 53°C to 68°C (T_0.5_ = 58.6°C), similar to noxious TRPV receptors in mammals. Monomeric and tetrameric ion channel structure predictions show reliable parallels with fruit fly dTRPA1, with structural uniqueness in ankyrin repeat domains, the channel selectivity filter, and potential TRP functional modulator regions. Overall, the finding of a member of *TRPA5* as a temperature-activated receptor illustrates the diversity of insect molecular heat detectors.

## Highlights

• Insect TRPs are a diverse gene family comprising molecular heat sensors
• *TRPA5s* were lost during the evolution of Diptera but are present in other orders
• *TRPA5B* encodes a highly thermosensitive receptor in the kissing bug
• Activity threshold and stimulus range suggest a role in noxious heat sensing

## Graphical abstract

**Figure:**
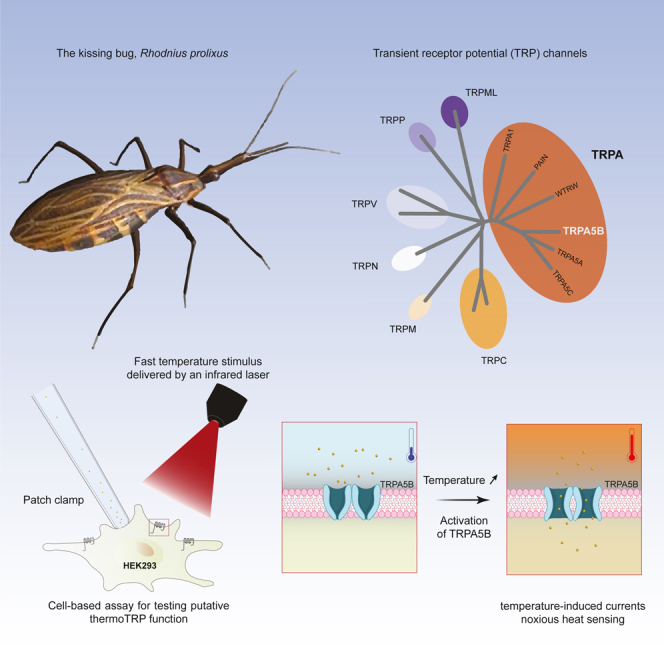

## Introduction

Animal thermosensation is critical for performance in fluctuating environments. Changes in environmental temperature are transduced by the sensory system as part of physiological feedback controlling responses such as metabolic homeostasis, feeding, finding suitable habitats, and extreme-heat avoidance.^1^^,^^2^ At the molecular level, thermal perception is mediated by the temperature-dependent activation of specific cold- and heat-activated receptors.^3^^,^^4^ Although families such as ionotropic receptors (IRs) and gustatory receptors (GRs) have been linked to peripheral innocuous thermosensation in insects,^3^^,^^4^^,^^5^^,^^6^ the transient receptor potential (TRP) receptor family encodes the greatest diversity of thermosensitive channels. TRP receptors are remarkably diverse (TRPA, TRPC, TRPN, TRPM, TRPML, and TRPV) and play salient roles as polymodal ion channels responding to chemical, mechanical, and thermal stimuli.^7^^,^^8^^,^^9^^,^^10^^,^^11^^,^^12^

**Figure 1:**
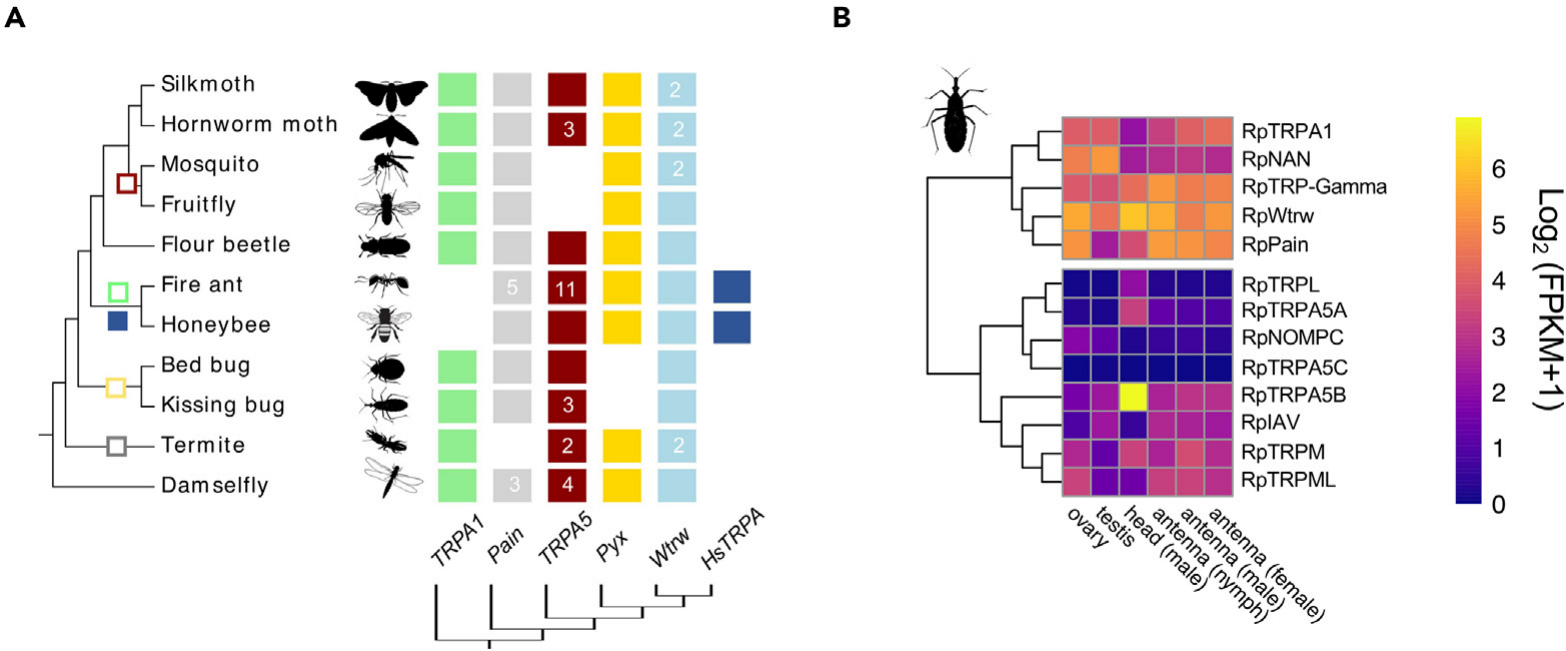
Phylogeny and expression of *Rhodnius prolixus* TRPs (A) Phylogenetic reconstruction of the ankyrin TRP (TRPA) channel subfamilies in representative insect species. TRPA5 channels are present across insect orders but absent from dipteran genomes (see also Table S1; Figures S1 and S2). Gene abbreviations: *Painless (Pain), Pyrexia (Pyx), Waterwitch (Wtrw*), *TRPA Hymenoptera-specific* (*HsTRPA*). Silkmoth, *Bombyx mori*; Hornworm moth, *Manduca sexta*; Mosquito, *Anopheles gambiae*; Fruit fly, *Drosophila melanogaster*; Flour beetle, *Tribolium castaneum*; Fire ant, *Solenopsis invicta*; Honeybee, *Apis mellifera*; Bed bug, *Cimex lectularis;* Kissing bug*, Rhodnius prolixus*; Termite, *Zootermopsis nevadensis*; Bluetail Damselfly, *Ischnura elegans.* Gene gain: filled square; gene loss: empty square. Numbers within squares indicate gene number when different from 1. (B) TRP genes in *R. prolixus* and their relative expression levels across tissues in compiled transcriptomic data (*see*STAR Methods). Heat maps compare the expression levels across tissues and developmental stages. Expression levels are represented as Log_2_ FPKM +1 and depicted with a gradient color scale. Gene models are based on genomic annotations,^36^ and *de novo* transcriptome assembly^45^ (see also Tables S2 and S3; Figure S3).

Mammalian TRP channels involved in temperature detection (thermoTRPs) belong to the TRPV, TRPA, and TRPM subfamilies and are activated by temperatures from noxious cold to noxious heat^4^^,^^9^^,^^13^^,^^14^^,^^15^^,^^16^ (Table 1 and references therein). In invertebrate species examined thus far, molecular and functional studies have uncovered several members of the ankyrin TRPA subfamily to function as thermoTRPs, including *Painless*, *Pyrexia*, *TRPA1*, and *HsTRPA* (Hymenoptera-specific) (Figures 1A and S1).^11^^,^^12^ For instance, in the fruit fly *Drosophila melanogaster*, *Painless*, *Pyrexia*, and *dTRPA1* isoforms A, B, and D encode receptors that exhibit distinct biophysical properties, cellular expression patterns, and temperature activation thresholds ranging from 19°C to 46°C.^17^^,^^18^^,^^19^^,^^20^^,^^21^^,^^22^^,^^23^^,^^24^ TRPA1 is also a heat-activated TRP sensor in *Anopheles gambiae* (25°C–37°C), and other mosquitos,^25^^,^^26^ playing a key role in tuning heat-seeking behavior. Outside the Diptera, TRPA1 has been characterized as a heat-sensitive channel in other insects as it is known to regulate the induction of embryonic diapause in *Bombyx mori* at temperatures above 21°C.^27^ The subfamily *Waterwitch* (*Wtrw*) includes receptors responding to stimuli in different modalities, from ancestral hygrosensation found in fruit flies^20^ to derived heat sensing exhibited by hymenopterans and mediated by the HsTRPA subfamily, which diverged following a duplication from *Wtrw*.^12^ Thus, despite the loss of TRPA1 in Hymenoptera, in the honeybee, *Apis mellifera* Am-HsTRPA responds to temperatures around 34°C, and, in the fire ant *Solenopsis invicta*, Si-HsTRPA is activated in the range 28°C–37°C, whereas, in the parasitoid wasp *Nasonia vitripennis*, Nv-HsTRPA activates in response to small temperature differences in the range 8°C–44°C regardless of initial temperatures.^28^^,^^29^ Notably, the insect TRP ankyrin family has an additional subfamily of unknown function, TRPA5, which is absent from the fruit fly genome yet found across several other orders of insects^11^ (Figure 1A).

**Table 1:** Transient receptor channels involved in thermosensation in invertebrates and vertebrates

TRP channel	Alternative name	Organism	Tissue distribution	TRP subfamily	Thermal sensitivity^a^	Activity range	Entropy change (ΔS) cal/mol∗K	Activation enthalpy (ΔH) kcal/mol	Q10^b^	Reference
*TRPV1*	Vr1	*Rattus norvegicus* (rat), *Homo sapiens* (human)	nociceptor neurons	Vanilloid	≥42°C (40–45), T(_0.5_) = 51°C	40.9°C-55.8 °C^c^	260 (at 30mV)	90–100	16.8, 20	Caterina et al.^13^, Yao et al.^52^, Tominaga, et al.^54^, Liu et al.^55^, Cheng et al.^65^
*TRPV1(l)*	TRPV1- long	*Desmodus rotundus* (vampire bat)	Dorsal root ganglion (DRG) and trigeminal ganglion (TG) neurons	Vanilloid	40°C	ND	ND	ND	12	Gracheva et al.^14^
*TRPV1(s)*	TRPV1- short	*Desmodus rotundus* (vampire bat)	TG neurons	Vanilloid	30°C	ND	ND	ND	5	Gracheva et al.^14^
*TRPV2*	Vrl2	*Rattus norvegicus* (rat), *Homo sapiens* (human)	nociceptor neurons, brain, spinal cord	Vanilloid	≥52°C (50°C–53°C)	ND	586	200	20.6	Caterina et al.^15^
*hTrpv3*	Vrl3	*Homo sapiens* (human)	skin keratinocytes	Vanilloid	≥33°C (34°C–38°C)	33°C–50°C (Max 41°C–47°C)	ND	ND	6.62	Peier et al.^123^, Smith et al.^124^, Xu et al.^125^
*hTrpv4*	OTRPC4, VR-OAC, Trp12, Vrl2	*Homo sapiens* (human)	skin and dorsal root ganglion neurons, kidney, brain, liver, heart, hypothalamus	Vanilloid	∼27°C–34°C	ND	ND	ND	9.9 ± 3.8	GÜler et al.^73^, Watanabe et al.^126^
*TRPM8*	CMR1	*Homo sapiens* (human)	DRG neurons, various organs	Melastatin	≤23°C–25°C	0°C-25°C	−384	−112	24	Brauchi et al.^70^, McKemy et al.^127^
*TRPM3*		*Mus musculus* (mouse)	DRG and TG sensory neurons	Melastatin	≥43°C	ND	ND	ND	7.2	Vriens et al.^16^
*TRPC5*		*Mus musculus* (mouse), *Homo sapiens* (human)	DRG neurons	Canonical	<37°C to >25°C	ND	ND	−40	ND	Zimmermann et al.^128^
*Anktm1*	TRPA1	*Mus musculus* (mouse), *Homo sapiens* (human)	DRG neurons	Ankyrin	≤17°C	0°C–17°C	−140	−40	6	Story et al.^129^
*Chicken TRPA1*	TRPA1	*Gallus gallus domesticus* (chicken)	DRG neurons	Ankyrin	39.4°C	ND	ND	ND	ND	Saito et al.^130^
*xtTRPA1*	TRPA1	*Xenopus tropicalis* (Western clawed frog)	DRG neurons	Ankyrin	39.7°C	ND	ND	ND	59.24 ± 18	Ohkita et al.^131^
*xlTRPA1*	TRPA1	*Xenopus laevis* (African clawed frog)	DRG neurons	Ankyrin	36.2 ± 0.4°C	ND	ND	ND	ND	Saito et al.^132^
snTRPA1	TRPA1	*Crotalus atrox* (rattlesnake)	TG neurons	Ankyrin	27.6°C	ND	ND	ND	13.7	Gracheva et al.^133^
python TRPA1	TRPA1	*Python regius* (python)	TG neurons	Ankyrin	32.7°C	ND	ND	ND	ND	Gracheva et al.^133^
boa TRPA1	TRPA1	*Corallus hortulanus* (boa)	TG neurons	Ankyrin	29.6°C	ND	ND	ND	ND	Gracheva et al.^133^
ratsnake TRPA1	TRPA1	*Elaphe obsoleta lindheimeri* (rat snake)	TG neurons	Ankyrin	37.2°C	ND	ND	ND	8.8	Gracheva et al.^133^
Anole TRPA1	TRPA1	*Anolis carolinensis* (green anole)	TG neurons	Ankyrin	33.9°C	ND	ND	ND	45.71 ± 6	Saito et al.^134^
*A. allogus* TRPA1	TRPA1	*Anolis allogus* (spanish flag anole)	Dorsal skin	Ankyrin	33.5 ± 0.7°C	ND	ND	ND	ND	Akashi et al.^135^
*A. homolechis TRPA1*	TRPA1	*Anolis homolechis* (Habana anole)	Dorsal skin	Ankyrin	36.4 ± 0.8°C	ND	ND	ND	ND	Akashi et al.^135^
*A. sagrei TRPA1*	TRPA1	*Anolis sagrei* (brown anole)	Dorsal skin	Ankyrin	33.5 ± 0.7°C	ND	ND	ND	ND	Akashi et al.^135^
*axTRPA1*	TRPA1	*Ambystoma mexicanum* (axolotl)	Brain, lung, heart, stomach	Ankyrin	39.7 ± 1.0°C	ND	ND	ND	ND	Oda et al.^136^
*zTRPA1b*	TRPA1	*Danio rerio* (zebrafish)	Sensory neurons innervating skin and cranial sensory ganglia	Ankyrin	variable <10°C (cold); >25°C (heat)	ND	ND	ND	8.2 ± 0.6	Oda et al.^137^
*olTRPA1*	TRPA1	*Oryzias latipes* (medaka)		Ankyrin	variable	ND	ND	ND	ND	Oda et al.^138^
*trTRPA1*	TRPA1	*Takifugu rubripes* (pufferfish)		Ankyrin	variable <8°C (cold); >25°C (heat)	ND	ND	ND	ND	Oda et al.^139^
*hTrpv4*	OTRPC4, VR-OAC, Trp12, Vrl2	*Homo sapiens* (human)	skin and dorsal root ganglion neurons	Vanilloid	∼27°C–34°C	ND	ND	ND	9.9 ± 3.8	GÜler et al.^73^, Watanabe et al.^126^
*ceTRPA*		*Caenorhabditis elegans* (nematode)	neurons, muscle, intestine, and epithelial cells		≤17°C	ND	ND	ND	ND	Laursen et al.^83^
*Painless*	dAnktm1	*Drosophila melanogaster* (fruit fly)	Peripheral neurons larval epidermis	Ankyrin	∼39°C–42°C	ND	ND	ND	ND	Tracey et al.^17^ Sokabe et al.^96^
*Pyrexia*	Pyx-PA and Pyx-PB	*Drosophila melanogaster* (fruit fly)	embryos: peripheral nerves, central nerves, multidendritic neurons in larval epidermis; adults: sensory neurons around bristle eyes, bristle neurons along thorax, neurons in maxillary palps, proboscis, antennae	Ankyrin	≥40°C	ND	ND	ND	Pyx-PA 18.145; Pyx-PB 15.329	Lee et al.^18^, Neely et al.^63^
*dTRPA1(A)*	TrpA1-RI (Prom B, ex10a), dTrpA1, dANKTM1, DmTRPA1	*Drosophila melanogaster* (fruit fly)	larval sensory neurons, adult proboscis	Ankyrin	27°C-29°C	ND	ND	ND	9	Kang et al.^19^, Hamada et al.^21^, Rosenzweig et al.^64^, Viswanath et al.^140^
*dTRPA1(D)*	TrpA1-RG (Prom A, ex10a), dTrpA1, dANKTM1, DmTRPA1	*Drosophila melanogaster* (fruit fly)	larval nociceptors	Ankyrin	≥46°C, T(0.1) = 43°C^c^, T(0.5) = 53.5 °C^c^	43-60 °C^c^	211^c^	69^c^	23,58^c^	Zhong et al.^22^
*AgTRPA1*		*Anopheles gambiae* (African malaria mosquito)	antennae, head	Ankyrin	25.2°C	ND	ND	ND	ND	Wang et al.^25^
*AsTRPA1(A)*		*Anopheles stephensi* (Asian malaria mosquito*)*	antennae, head	Ankyrin	30.3 ± 0.9°C	ND	ND	ND	14,5	Li et al.^141^
*AaTRPA1(B)*		*Aedes aegypti* (yellow fever mosquito)	antennae, head	Ankyrin	32 ± 0.8°C	ND	ND	ND	20,7	Li et al.^141^
*CpTRPA1(A)*		*Culex pipiens pallens* (northern house mosquito)	antennae, head	Ankyrin	21.8 ± 0.7°C	ND	ND	ND	61,2	Li et al.^141^
*BmTRPA1*		*Bombyx mori* (silk moth)	epidermlal non-neural cells of various tissues	Ankyrin	21.6°C	21.6°C–43.5°C	ND	ND	20,5	Saito et al.^130^
*HsTRPA*	Am-HsTRPA	*Apis mellifera* (honeybee)	brain, leg, proboscis, antennae	Ankyrin	33.9 ± 0.6°C	ND	ND	ND	17.2 ± 4.0	Kohno et al.^28^
*RpTRPA5B*^c^		*Rhodnius prolixus* (kissing bug)	head, rostrum, legs, antennae, thorax, abdomen	Ankyrin	T(0.1) = 53°C; T(0.5) = 58.6°C	50°C-68°C	216	72	25	c

a activation threshold temperature as determined in heterologous expression systems.

b Q10 is the fold current increase over 10°C increase as a measure of sensitivity; the higher Q10 value, the more sensitivity to heat exhibited by the TRP channel.

c experimental values determined in this study.

Here, we de-orphanize and characterize an ankyrin TRPA5 ion channel from the triatomine bug, *Rhodnius prolixus*. Long used as a model organism in studies of insect development and physiology,^30^^,^^31^ *R. prolixus* (Hemiptera; Reduviidae: Triatominae) has become increasingly relevant for molecular and functional studies. This is primarily explained by its long-term medical and societal impact as a hematophagous vector of *Trypanosoma cruzi*, the causative agent of Chagas disease.^32^ In Latin America, where the disease has traditionally affected the health of millions of people, vector transmission is estimated to cause around 30,000 cases and 12,000 deaths annually.^33^^,^^34^ Although transmission rates have decreased over the last five decades, the progressive adaptation of wild triatomine vector species to domestic environments and the ongoing challenge of vector transmission to human populations, coupled with the lack of vaccines, constitute a significant and overlooked general public health concern.^34^^,^^35^ Extensive long-term efforts toward decoding the sensory ecology of triatomines^30^^,^^36^^,^^37^ have identified olfactory, thermal, and environmentally mediated cues as well as the neuroethology underlying its complex host-seeking behavior.^37^^,^^38^^,^^39^^,^^40^^,^^41^^,^^42^^,^^43^^,^^44^^,^^45^^,^^46^ Moreover, the annotated *R. prolixus* genome^36^ and recent transcriptomic studies^45^^,^^46^^,^^47^ provide detailed profiles of candidate sensory receptor genes, including olfactory, ionotropic, pickpocket, and TRP receptors that can be used to probe the genetic basis of sensory traits.^48^^,^^49^

By leveraging genomic and transcriptomic resources available for *R. prolixus* along with molecular, structural modeling and functional approaches, we characterize a broadly expressed TRPA5 ion channel. The biophysical properties of the ion channel demonstrate that *RpTRPA5B* encodes an ankyrin type of heat-activated TRP receptor responding to noxious temperatures *in vitro*. Analyses of predicted structures reveal that the channel displays shared conserved structural domains with other ankyrin TRPs combined with unique features among the ankyrin family.

Our results shed light on the molecular function of a TRPA5 channel as a receptor activated by a wide range of thermal stimuli, which likely, together with other thermoTRPs, contributes to the extreme sensitivity of *Rhodnius* in detecting heat across various behavioral contexts.

## Results

### Genomic and phylogenetic placement of Rhodnius ankyrin TRPs

To begin investigating the molecular basis of thermosensation in *Rhodnius prolixus*, we reanalyzed the genome annotation (version RproC3.3) complemented with transcriptomic resources (see STAR Methods) to gain insights into gene variation and genomic architecture within the *R. prolixus* TRP ankyrin family. The genome of *R.* *prolixus*^36^ and available transcriptomes^45^^,^^47^^,^^50^ of triatomines, and additional surveyed hemipteran species (Figures 1A and S1; Table S1), harbor members of four TRPA subfamilies. All surveyed heteropteran species appear to lack an ortholog to *Pyrexia* (*Pyx*) TRP^36^^,^^50^ but possess one gene copy of three canonical ankyrin TRP genes: *Waterwitch* (*Wtrw*), *TRPA1*, and *Painless* (*Pain*) (Figures 1A and S1). Three *TRPA5* transcripts were previously described in *R. prolixus*.^45^ We updated the genome annotation of the reference assembly using transcriptomic datasets and found that *TRPA5A* (RPRC001596) and *TRPA5B* (RPRC001597) map to different genomic locations on a single scaffold and consist of two physically close tandem-duplicate loci, whereas *TRPA5C* (RPRC000570) maps to a distinct scaffold. Intrigued by the finding of multiple *TRPA5* gene copies, we performed an extensive *TRPA5* gene search across annotated genomic and transcriptomic datasets available for the insect orders Anoplura, Diptera, Coleoptera, Hemiptera, Hymenoptera, Isoptera, Lepidoptera, Odonata, and Thysanoptera. Our phylogenetic reconstruction shows that the TRPA5 ankyrin subfamily is completely absent in all surveyed dipteran genomes (Figure S2), but TRPA5 orthologues are present at least in the orders Lepidoptera, Coleoptera, Hymenoptera, Hemiptera, Isoptera, and Odonata (Figures 1A and S2).

### Transcriptomic and quantitative expression of *TRPA5*

We next analyzed RNA sequencing (RNA-Seq) raw data to assess the expression profile of *TRPAs* for *Rhodnius prolixus* (Figure 1B). *TRPA1*, *Waterwitch*, and *Painless* appear broadly and highly expressed. The three *TRPA5* genes differed more in their expression pattern: RpTRPA5A and RpTRPA5C mRNAs are expressed at low detection thresholds, and RpTRPA5B mRNA is moderately abundant across the range of surveyed tissues, including male head and adult antennae (Figure 1B). Intrigued by high head expression and using complementary analyses by quantitative PCR, we confirmed that RpTRPA5B is also highly expressed in female heads, and ubiquitously expressed in the thorax, abdomen, rostrum, and legs (Figures 1B and S3), which directed our choice toward this TRP gene for functional analyses on this channel subfamily.

### Validation of a functional assay using whole-cell patch clamp and optical heat-pulse delivery

**Figure 2:**
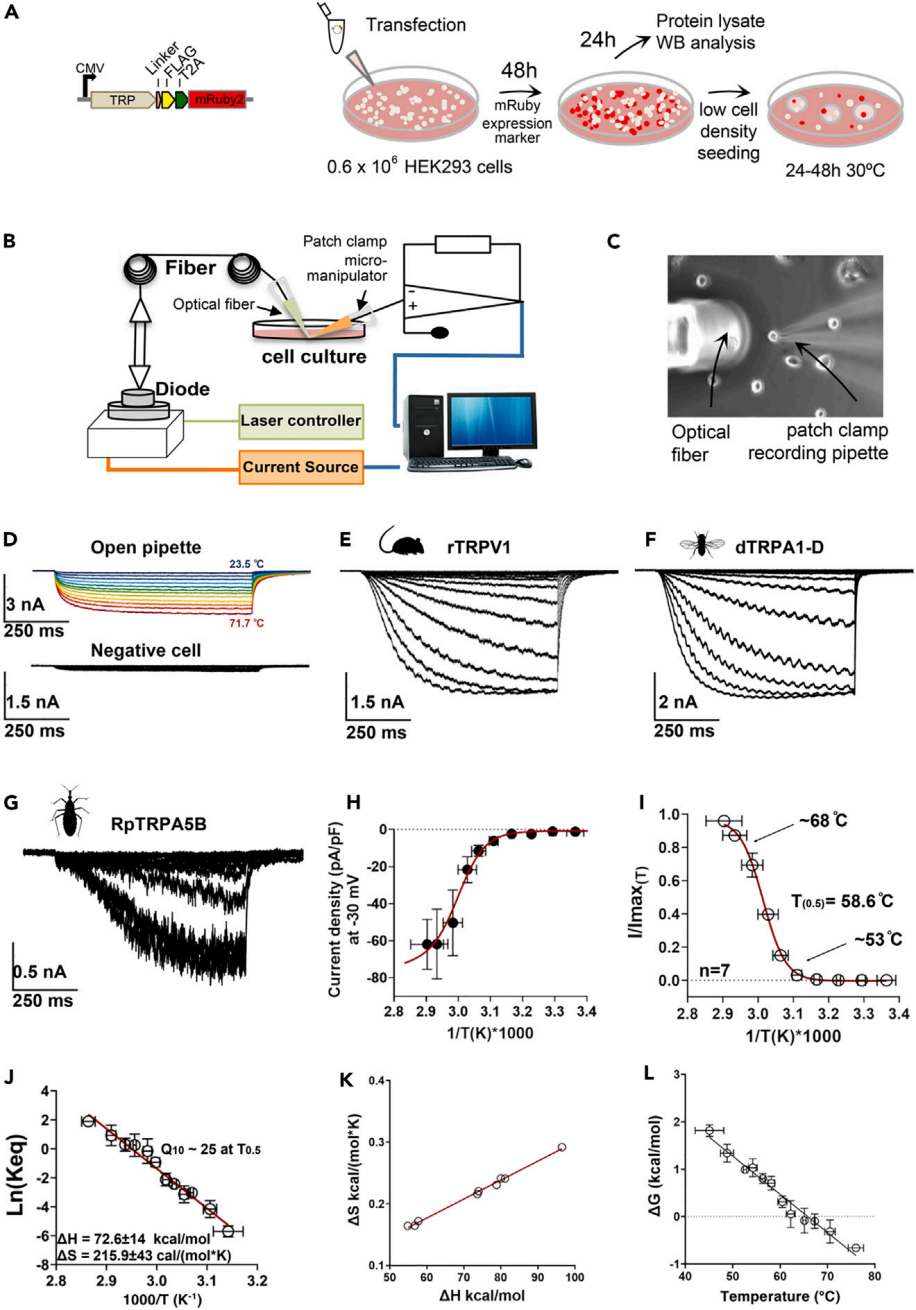
Thermodynamics of RpTRPA5B temperature-activated currents (A–C) Experimental workflow. (A) Each TRP channel subcloned in the pFRT-TO-FLAG-T2A-mRuby2 expression cassette^51^^,^^100^ was transfected in HEK293T cells seeded at low density and incubated at 37°C for 48 h. Cells were then prepared for patch-clamp recording by seeding in a 30-mm^2^ culture dish overlaid with round glass cover slips and incubated at 30°C. (B) Electrophysiology recordings took place after 24–48 h using an optical fiber-based setup adapted after Yao et al. 2010,^52^ designed to couple manual patch-clamp recordings with fiber optics as a way to provide controllable optical and thermal stimulations to individual cells expressing candidate thermosensitive receptor proteins. The setup consists of a fiber launch system combining a high-power optical fiber tuned to near-infrared wavelengths (λc = 1,460 nm (+/−20 nm), Po = 4.8 W), a visible alignment laser (red), and a laser diode controller, forming a PID control loop using the patch-clamp current as the feedback signal. (C) During the experiment, a laser spot is aligned with one single patched cell (see Figure S6) stably expressing the membrane receptor protein of interest in the coverslip placed in the recording chamber. (D) *Upper panel*, current traces through the open patch-clamp pipette in response to temperature calibration steps from room temperature up to 71°C elicited by increments in the IR laser voltage input (see STAR Methods). Each 700 ms voltage pulse is represented in different colors for the different temperatures calculated from the open pipette currents. *Lower panel*, representative recording of non-transfected cells; these cells did not show robust temperature-elicited currents, like negative cells on the recording plate. (E) Whole-cell currents evoked by temperature steps from HEK293T cells expressing rat TRPV1 (heat-activated mammalian vanilloid thermoTRP); cells were held at −30 mV during the recording. (F) Whole-cell currents evoked by temperature steps from HEK293T cells expressing dTRPA1-D (holding potential of −30 mV). The sinusoidal pattern observed within the current curves is inherent to the cyclic modulation of the laser’s rapid “on-off” cycles. (G) Whole-cell currents evoked by temperature steps in HEK293T cells expressing RpTRPA5B; cells were held at −30 mV. (H) Current-temperature relationship for RpTRPA5B whole-cell current was normalized by cell membrane capacitance (current density); the red line corresponds to a modified Boltzmann function that includes the leak and unitary current temperature dependence (see STAR Methods). (I) Fraction of RpTRPA5B channels in the open state (open probability, P_o_) as a function of the temperature. The Po vs. 1/T was fitted to a Boltzman function with the midpoint of activation (T_0.5_) reached at 58.6°C. (J) van’t Hoff plot estimates of RpTRPA5B with an activation enthalpy of the endothermic transition at 92 kcal/mol and an entropic change associated with the temperature activation process at 274 cal/mol∗K at −30 mV.^52^ (K) Coupling between enthalpic (ΔH) and entropic (ΔS) changes for each one of the experiments recorded. (L) Free energy (ΔG) associated with the activation process as a function of temperature for RpTRP5AB channels. The receptor activation is associated with small free energy changes, as reported before for other families of mammalian thermoTRP receptors. ΔG was calculated as -RT∗ln(Keq).^72^ Data are represented as mean ± standard error.

In order to demonstrate the potential role of candidate TRPA5B as a thermosensitive ion channel, we transiently expressed a bicistronic T2A-fluorescent marker cassette^51^ together with the candidate TRP channel, which localized well to the plasma membrane (Figures 2A and S4) and optimized an *in vitro* cell-based workflow to record temperature-elicited currents from HEK293T cells under whole-cell patch-clamp configuration (Figures 2B and 2C). Fast temperature stimulus was delivered by coupling an infrared laser diode to fiber optic after Yao et al.^52^ A proportional-integral-derivative (PID) controller was used to keep the temperature stable along the duration of the pulse (700 ms), turning on and off the diode using the open pipette current trace as feedback for the PID controller, and calculating the steady-state parameters of activation from the current at the end of the 700 ms temperature pulse. The magnitude of ionic current changes through the open patch-clamp pipette was used to calculate the temperature changes associated with the different laser intensities (Figures 2B, S5, and S6). During this calibration, the laser voltage input and the series of pulses necessary to reach the desired temperatures are recorded, and this file is later played back to the diode. The patch-clamp recording pipette is positioned in the exact same position relative to the optic fiber during the calibration (Figure S6), and each cell recorded has its own calibration file (see STAR Methods). To validate this modified infrared (IR) patch-clamp system and expression cassette, we first transiently expressed two control thermoTRPs, the rat TRPV1 (rTRPV1) and fruit fly TRPA1 isoform D (dTRPA1-D) (Figures 2D–2F and S5). At the molecular level, both rTRPV1 and dTRPA1-D formed expected homotetrameric structures (Figure S4).

### Full current activation response profiles at high temperatures for rTRPV1 and dTRPA1-D

Current pulses were set to result in temperature increments at the cell membrane in the range of 23.5°C–71.7°C, at a holding membrane potential of −30 mV (Figure 2D). This voltage magnitude provides a driving force big enough to resolve the ionic currents and minimizes a potential influence of the membrane voltage over the temperature activation process.^52^ A similar laser stimulation protocol led to marginal whole-cell current changes in non-transfected cells (Figures 2D, S5A, and S5B). Compared to non-transfected cells, we then observed a strong increase in the current amplitude of cells expressing rTRPV1 (Figures 2E, S5C, and S5D) with an enthalpy change associated with the activation of 88.3 ± 9.4 kcal/mol, which is comparable to the published enthalpy values obtained using millisecond temperature jumps of ΔH = 85 kcal/mol for rTPRV1^52^ (see Table 1).^13^^,^^52^^,^^53^ The temperature values as shown in Figure S5 for rTRPV1 align with those reported in earlier studies for this channel. The published threshold of activation for rTRPV1 is in the range 40°C–42°C and corresponds to the temperature at which the first observable currents were detected.^13^^,^^54^^,^^55^ Hence, the channel emerging temperature recorded in our setup is at 40.9°C when the first activation currents emerge over the dotted line (Figure S5D). We calculated values of rTRPV1 T_0.1_ (i.e., the temperature for which there is a probability for 10% of channels to be open) at 45.3°C (−30mV), and T = _0.5_ at 51.6°C (−30mV), consistently with values reported by Yao et al.^52^ (T_0.5_ = 51°C, −60mV). A temperature-induced activation response was also observed for the heat-activated fruit fly channel, dTRPA1-D, for which in our more precise setup, at 46.3°C,^22^ the open probability (Po) of the channel is about 10% (Po = 0.1), corroborating a higher-than-ambient activation temperature >42°C.^22^ Assuming complete activation of this channel (Po = 1) by temperature, which was not measured in previous studies due to limitations in the maximum temperature to which the dTRPA1-D channel could be subjected, the activation process is characterized by an enthalpy change ΔH = 68.7 ± 13.1 kcal/mol and T_0.5_ = 53.5°C (Figures 2F, S5E, and S5F).

### Controlled temperature-dependent biophysical properties of RpTRPA5B

RpTRPA5B similarly assembled as a membrane-bound homotetramer when expressed in HEK293T cells (Figure S4). When holding the membrane potential at −30mV in patched mRuby2-expressing cells transfected with RpTRPA5B, whole-cell currents were evoked by temperature steps a little above 50°C (Figures 2G and 2I). The average temperature for the activation “threshold” was 53°C, defined as Po = 0.1 calculated from the van’t Hoff plots. The channel opening appeared to saturate at 68°C (Po = 0.9) (Figure 2I), with a T_0.5_ = 58.6°C. The current density versus temperature relationship (Figure 2H) indicates that the opening of RpTRPA5B involves an activation enthalpy (ΔH) of approximately 72.6 ± 14 kcal/mol (Figure 2J). The large entropy value (Figures 2J and 2K) further indicates that the channel transits between a highly ordered closed state and a strongly disordered open configuration, close to activation enthalpy for TRPV1 (ΔH 101 ± 4 kcal/mol at −60 mV).^52^ Based on van’t Hoff plots thermodynamic parameters, we further calculated a Q_10_ value of ∼25, which is in the range of characterized noxious vertebrate receptors (rTRPV1 Q_10_ = 16.8; rTRPV2 Q_10_ = 20.6) and the invertebrate fruit fly Pyrexia (Q_10_ = 18.2) (*see also* Table 1).

### Insights from Rhodnius TRPA5B monomeric and tetrameric structure predictions

**Figure 3:**
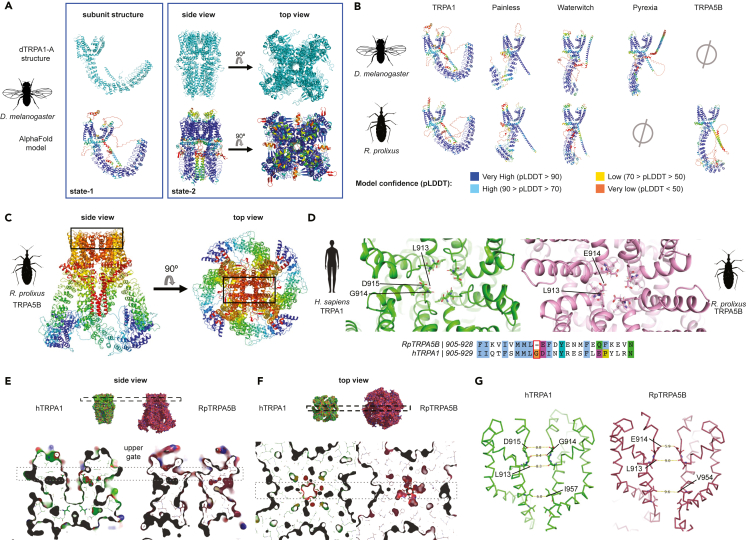
Monomeric and tetrameric assemblies of RpTRPA5B channels modeled using AlphaFold, after validation with dTRPA1 structure (A–D) (A) *(left panel) Upper row*, cartoon representation of chain A in tetramer of dTRPA1 in state 1 (PDB: 7YKR). The fold of a monomer in the experimentally determined structure of the dTRPA1 tetramer is very similar to the fold of an AlphaFold model of a single dTRPA1 monomer (*bottom row*). AlphaFold model colored from red to blue according to pLDDT confidence scores as shown in (B). The low-confidence regions (red) are not resolved in the reported structure and are likely to be intrinsically disordered. (*Right panel) Upper row*, experimentally determined structure of dTRPA1 in state 2 (PDB: 7YKS). *Bottom row*, tetrameric AlphaFold model of dTRPA1 depicted as cartoons colored from red to blue according to confidence scores as in (B). The N- and C-terminal regions, which are not resolved in 7YKS, were excluded in the prediction. Only the last five of the 17 ankyrin repeats (AR12-16) are visible in the structure and overall regions with low confidence in the model (red-yellow) are not resolved in the structure. (B) Monomers of *Drosophila* and *Rhodnius* TRPAs colored by pLDDT score from the AlphaFold modeling. (C) Tetrameric model of RpTRPA5B, colored as chain bows (N terminus, blue; C terminus, red). The black box indicates the location of the pore and selectivity filter shown in (D). (D) Top view of the selectivity filter of the pore of hTRPA1 (*left*: human TRPA1, PDB: 6V9Y) and RpTRPA5B (*right*). Three important residues^61^ – L913, G914, and D915, are marked in hTRPA1. The equivalent residues L913 and E914 are marked in RpTRPA5B, and G914 absent in RpTRPA5B is highlighted in the sequence alignment, together with additional residue changes adjacent to the selectivity filter. (E and F) Comparison of the pore in hTRPA1 and model of RpTRPA5B indicates a closed upper gate and an open lower gate in the RpTRPA5B model. E. *(upper row)* Surface representation of hTRPA1 (green; PDB: 6V9Y) and RpTRPA5B (pink), side view. The dashed box indicates the location of the upper gate toward the outside of the cell. *(lower row)* Slab along the pore through the transmembrane domain of the hTRPA1 structure and RpTRPA5B model. F. *(upper row)* Top view of hTRPA1 and RpTRPA5B shown in E. *(lower row)* The slab is perpendicular to the pore at the level of the upper gate shown in E. The dashed box indicates the location of slab in (E). (G) Distances between corresponding residues in the upper and lower gate in structures of hTRPA1 and the model of RpTRPA5B, shown as sticks.

To visualize and compare structural features between TRPA5B and other ankyrin TRP homologs, we used AlphaFold 2.0 to generate models without a structural template.^56^^,^^57^^,^^58^ This approach was first reliably validated by a comparison to the recently reported structure of *Drosophila melanogaster* dTRPA1-A in state 1, which confirmed all distinct predicted features in the monomeric model of dTRPA1, including the interfacial helix and the interaction between ankyrin repeat (AR) 12 and the region C-terminal of the coiled-coil helix (Figure 3A).^59^ Although some of the helices in the monomeric model are oriented in unrealistic directions owing to missing constraints of the other monomers and the interactions that would force the C terminus into the coiled-coil, their reliable secondary structure provides a first meaningful comparison of secondary structural elements and the general fold between proteins from different ankyrin TRP subfamilies.

All *Rhodnius* and *Drosophila* ankyrin TRP monomeric structures were then modeled following the same approach, supporting highly reliable predictions for orthologues (Figures 3B and S7) and expected structural similarities with the cryoelectron microscopy (cryo-EM) structures of *Drosophila* dTRPA1 and human hTRPA1,^60^ including the N-terminal AR domain, six transmembrane α-helices (S1-S6), and a region corresponding to the allosteric nexus of hTRPA1 connecting the AR domain and the transmembrane region.^60^ In addition, the monomeric N termini show an overall conserved stacking of the ARs (Figures 3B and S7)—*albeit* with clade-specific breaks and numbers of repeats (Table S5). The C-terminal regions feature at least one α-helix, which, together with the corresponding helices from the other subunits, most likely form a coiled-coil in the tetramers as seen in the solved TRPA1 structures.

We next generated a tetrameric model of RpTRPA5B to examine the predicted stable state of the pore and selectivity filter (Figures 3D–3G), following supporting evidence that an AlphaFold tetrameric model of dTRPA1 generated without a structural template proved highly comparable to the corresponding resolved structure of dTRPA1 in state 2 including the transmembrane domain, last part of the ankyrin repeat domain (ARD) (ARD12–16), and coiled-coil regions (Figure 3A, see Figure S8). For this, the root-mean-square deviation (RMSD) of the 20 Cα atoms of residues in the upper and lower gate of the pore was low (0.328 Å) (Figure S8D; Table S6) with the sole main deviation found in the side chain of Glu982 (Figure S9D). However, in the selectivity filter in the upper gate of the RpTRPA5B tetramer model (Figures 3C and 3D), one glycine suggested to be important in gating^61^ (Gly914 in hTRPA1, PDB:6V9Y) is absent in RpTRPA5B (Figure 3D), but conserved in most other TRPAs or substituted for serine or threonine in most other non-hemipteran TRPA5 proteins (see Figure S2). Other adjacent residue changes are located in this region, and, while Leu913 and Glu914 in RpTRPA5B form a shorter pore loop, they maintain the same overall locations with Leu913 and Asp915 in hTRPA1. Finally, an interesting last feature is the pore of the RpTRPA5B model, which appears to be open at the lower gate and closed at the upper gate (Figures 3E–3G and S9; Table S6), reversely to the hTRPA1 and dTRPA1 structures (PDB:6V9Y, 7YKS).

## Discussion

### *TRPA5* evolutionary dynamics and function support insect thermoTRP channel usage plasticity

Large-scale phylogenetic reconstructions combining TRP genes from 46 insect families spanning 9 major orders provided additional insights into the dynamic evolution of five insect TRPA ankyrin clades:^11^^,^^12^ *TRPA1*, *Painless*, *Waterwitch* (including *HsTRPA1*), *Pyrexia*, and *TRPA5* (Figures 1 and S1; Table S1). In addition to complex alternative splicing,^62^ remarkable group-specific expansions of *TRPA5* genes as observed in the fire ant, *Solenopsis invicta*,^29^ in the damselfly *Ischnura elegans*, the tobacco hornworm moth *Manduca sexta*, and several hemipteran species including the kissing bug, *Rhodnius prolixus*, seem to play a role in the TRPA5 clade diversification (Figures 1, S1, and S2). Whereas the TRPA5 clade is absent in all surveyed mosquitoes and flies (Diptera), reversely, *Pyrexia* genes, encoding a class of functional noxious heat receptors in fruit flies,^18^ are retained in all major insects except in hemipterans (Figure S1).^50^ Dynamic gene loss-gain among insect TRPA lineages together with experimental evidence of a hemipteran thermosensitive TRPA5 supports channel usage plasticity and convergence in noxious thermal activation range over millions of years of divergent evolution. Altogether, our findings illustrate an example of the resilience of invertebrate sensory systems via compensatory molecular sensors of environmental thermal detection.

In *Rhodnius*, *TRPA* expression (Figure 1B) matches the tissue distribution range of insect thermoreceptors such as the canonical fruit fly *TRPA1*, *Painless*, and *Pyrexia*.^27^^,^^28^^,^^29^^,^^63^ For instance, *Painless* is expressed along the entire epidermis in fruit fly larvae^17^ and *Pyrexia* is expressed in sensory structures more broadly, including adult mouth structures (maxillary palps and proboscis), adult bristle sensilla, thorax, and eyes, and larval body epidermis.^18^ Different dTRPA1 isoforms localize in different tissues, including brain neurons and blood capillaries (isoform A),^21^^,^^64^ larval nociceptive neurons in the central nervous system (brain, isoform D),^22^ and proboscis (isoform C).^23^ Fruit fly *Wtrw* (a humidity sensor) and mosquito *TRPA1* localize to specific antennal sensilla,^20^^,^^25^ and honeybee and fire ant *HsTRPA1* orthologues are both expressed broadly including in leg, antenna, head, and proboscis (honeybee),^28^ or antenna, leg, head, thorax, and abdomen (fire ant).^29^ Although all *Rhodnius TRPA* genes likely have physiological roles, including *RpTRPA5A*, and *RpTRPA5C*, we chose to focus first on *TRPA5B* as representative to start investigating the biophysical properties of TRPA5 channels, guided by transcriptomic and quantitative tissue expression analyses that placed *TRPA5B* as an interesting broadly expressed TRP found across the adult body of *Rhodnius* (Figures 1B and S3), including tissues known to have potential thermosensing roles.

### TRPA5B is gated by noxious temperature stimuli *in vitro*

By delivering controlled optical heat pulses to HEK293T cells expressing TRP proteins under whole-cell voltage-clamp configuration at −30 mV, we first recapitulated reliably the biophysical properties associated with the thermal activation of two control thermoTRP channels, the rat rTRPV1 and the fruit fly dTRPA1-D (Figures 2 and S5). For the dTRPA1-D channel, our setup permitted extended biophysical characterization to obtain T_0.5_ = 53.5°C (ΔH = 69 kcal/mol, −30 mV), a widely accepted comparative measure of the temperature at which is achieved the probability of having 50% channels open, not calculated in earlier studies due to setup constraints.^22^ For rTRPV1, our data consistently recapitulated previously reported activation thresholds above 40°C (40.9°C, Figure S5D)^13^^,^^54^ and a value of T_0.5_ = 51.6°C (−30 mV) (Figure S5D),^52^^,^^55^ establishing a rigorous basis for temperature stimulus delivery to determine the biophysical properties of RpTRPA5B thermal activation. For RpTRPA5B, whole-cell currents were evoked above 50°C with the probability of having 10% channels open (Po = 0.1) at 53°C, a T_0.5_ = 58.6°C, and saturating currents were reached at 68°C, representing Po = 0.9 (Figure 2). High Q_10_ values for this channel at T_0.5_ were 25, comparable to values of Q_10_ 16–20 for rTRPV1^13^^,^^52^^,^^54^^,^^55^^,^^65^ and Q_10_ 20.6 for rTRPV2,^15^ Q_10_ 15–18 for fruit fly Pyx,^18^^,^^63^ and Q_10_ 23 for dTRPA1 (Figure 2; Table 1) whereas non-thermo-TRP channels typically have Q_10_ values below 3,^9^ supporting that RpTRPA5B is gated by temperature stimuli.

The relatively large observed enthalpy change (ΔH = 72.6 ± 14 kcal/mol at −30 mV) (Figure 3) required for the channel activation is also in line with a high sensitivity to temperature changes. Hence, high enthalpy changes of 100 kcal/mol and 88 kcal/mol are required to activate rTRPV1 at −60 mV^52^^,^^55^ and at −30 mV (Figure S5D), respectively, representing a 3-fold increase in temperature sensitivity at negative voltages^52^ compared to its activation in depolarizing conditions (30 kcal/mol at +60 mV). Similarly, RpTRPA5B showed a robust response at negative voltages (−30 mV) and almost no heat-elicited activity at depolarized potentials (>0 mV), supporting its dependence on temperature for activation. Large enthalpy changes ranging from 60 to 200 kcal/mol are also involved in the opening of other highly temperature-dependent channels including dTRPA1-D (Figure S5F), TRPM8, TRPV1, and TRPV2.^66^

The maximum Po value for TRPA5B’s thermal activation (Figure 3) is lower compared to the dTRPA1-D and rTRPV1 receptors, indicating that the activation kinetics of RpTRPA5B are lower than those of dTRPA1-D and rTRPV1, as can be appreciated by channel “noise” in the current traces,^67^ also reflected in the slower time course of activation of RpTRPA5B compared to the other thermosensors studied here. The maximum Po value reached experimentally in our study for rTRPV1 thermal activation is ∼1, in accordance with previous studies;^52^ however the maximum open probability of many ion channels is, in fact, typically lower than Po = 1.0. Low threshold T-type voltage-activated channels,^68^ NMDA receptors,^69^ and thermoTRP channels like TRPM8^70^ all show experimental maximal open probabilities lower than Po = 1.0. Likewise, the voltage-gated Shaker K^+^ channel has an activity plateau at ∼ Po = 0.7.^71^ All these receptors have a great influence on the excitability displayed by the cells expressing them.

TRP channels are allosteric receptors, meaning that each stimulus (temperature, voltage, agonist) is detected by an independent module able to activate either partially or fully the channel. In the case of RpTRPA5B, temperature seems to be a partial activator given that the open probability is significantly lower compared to the other receptors. This observation is not a predictor that the channel may be open fully in response to different types of stimuli, or combination thereof.^72^ Additionally, RpTRPA5B presents slow activation kinetics, compared with the other receptors studied under the same conditions. There have been previous reports of other highly sensitive thermosensor receptors with slow activation kinetics such as hTRPV3. This channel exhibits high-temperature sensitivity, comparable to its homolog hTRPV1, but presents slower kinetics.^66^ It is possible, like in the case of hTRPV3, that RpTRPA5B’s intrinsic molecular interactions influence the speed of the transitions between closed and open states, which would be interesting to test functionally, as it could potentially be explained by our observations of several structural differences in RpTRPA5B (Figure 3).

From a thermodynamic point of view, many TRP ion channels are modulated by temperature and thus can integrate voltage and temperature allosterically.^66^ To disentangle these two properties, we specifically established the temperature sensitivity of the channel directly from the van’t Hoff plot, and not from the potential influence of temperature itself on the voltage activation process. Since we do not have evidence that the receptor can be activated by voltage, this allowed us to establish the thermodynamics of the temperature activation, completely independently from other stimuli sources, clearly supporting that RpTRPA5B is directly activated by temperature as the sole stimulus and belongs to a restricted category of thermoTRPs.^8^^,^^72^ Finally, RpTRPA5B is also activated in a higher noxious range compared to known invertebrate thermoTRPs characterized thus far, including the fruit fly Painless and TRPA1 channels that mediate thermal nociceptive escape through larval mdIV neurons at temperatures above 40°C and 46°C, respectively,^17^ or Pyrexia channels that induce paralysis in adult flies upon exposure to 40°C.^18^ In summary, RpTRPA5B is a temperature-gated TRP receptor, with high-temperature sensitivity and activation responses to noxious heat stimuli *in vitro*. In mammals, only the vanilloid TRPV2 receptor contributes to such highly noxious (>52°C) heat sensing.^13^^,^^53^^,^^73^ The expression platform implemented here has the potential to open up comparative functional studies of TRPA5 orthologues as a potentially new class of highly noxious physiological sensors.

### Structural key features and insights into TRPA5B’s pore and ankyrin domain

All *Rhodnius* and *Drosophila* ankyrin TRP monomeric structures were modeled with AlphaFold after validation with dTRPA1 (Figure 3A). These models are snapshots of certain conformations and evidently do not reproduce the diversity of the different states a protein may adopt.^74^ Under default settings, AlphaFold provides a single state of otherwise highly dynamic proteins and is better at modeling backbone folding than individual sidechains. Taking these limitations into consideration, except for the orientation of the C-terminal helices, our results indicated that RpTRPA5B shares fundamental conserved regions and structural features with all modeled TRPA monomeric protein units (Figure 3B) and supported overall reliable secondary structure predictions close to tetrameric models and structures (Figure 3A). We further ran pairwise comparisons of the monomeric structures of RpTRPA5B and Pyrexia, alternatively retained in hemipteran and dipteran genomes, respectively (Figure 1). Interestingly, the two channels do not appear to occupy convergent homologous structural niches despite similarity in the ARD (Figure S7). Instead, RpTRPA5B appears to be generally closer in structure to Painless and Waterwitch in the transmembrane domain, while uniquely deviating in the specific details from modeled TRPA1 channels in the pore helices that flank the selectivity filter important for gating, and ARD previously suggested to contribute to thermosensitivity.^75^

To start addressing the potential relevance of these differences in the pore and ARD, we first validated a truncated tetrameric model of dTRPA1, which aligned very confidently to the channel released structure in state 2 (PDB ID 7YKS) with a low RMSD for the twenty residues forming the upper and lower gate in the conductivity pore, even when compared to alignments of the same channel in state 1, especially if the Cα are considered (Figure S8; Table S6). The modeling of the pore in RpTRPA5B is thus expected to be more reliable in the zones defined by the backbone and interestingly appears narrower than the closed conformation of hTRPA1,^61^ but with a wider lower gate (Figures 3D–3G and S9). In contrast to the lower gate, which is wider due to the position of helix S6, the constriction at the selectivity filter is mainly determined by the orientation of a side chain (Glu914), which is less reliable and likely to be more dynamic. In hTRPA1, Gly914 in hTRPA1 is suggested to be important in gating and lies in the location of the selectivity filter.^61^ It may seem peculiar that this highly conserved glycine residue is absent in RpTRPA5B; however a tetrameric model built simply inserting a Glycine residue in RpTRPA5B at that location causes significant deviations from the generic fold of TRPs including misplacement of helix S6 between adjacent monomeric units (Figure S10). These observations suggest that the expected folding is likely properly maintained owing to compensatory co-evolving adjacent changes in the protein sequence.

Typically formed by repeats of 31–33 residue protein motifs that occur in tandem arrangement, N-terminal ARDs are known to be critical for a number of physiological processes such as ligand binding or protein-protein interactions and occur in a wide range of proteins, including key sensory transducers of TRPA, TRPC, TRPN, and TRPV channel families.^76^ Compared to fruit fly Pyrexia and other insect TRPA channels, RpTRPA5B not only displays a higher number of ARs but also features longer loops, including between the third and the fourth ARs, within the fifth AR, and between the fifth and the sixth ARs, counting from the N terminus. Another interesting feature is the disruption in the AR stacking between the fifth and the sixth AR in both *Rhodnius* and *Drosophila* Painless, which is not seen in *Drosophila* Pyrexia and RpTRPA5B. Although the potential impact of this conserved difference is currently unknown, we note that this breaking point coincides with the resolved N-terminal end of the recently reported structure of dTRPA1-A in state 2, which has been suggested to represent a temperature-sensitized, pre-opened conformation of the channel.^59^

There is some evidence that the AR domain of some insect TRPAs may contribute to thermosensitivity, although more functional studies are needed prior to generalizing the role of ankyrin domains and establishing correlations linking variation therein with global and specific mechanistic and thermosensitive properties. In particular, the transfer of a part of the ARD from dTRPA1 (AR10-15) to hTRPA1 produced a heat-sensitive hTRPA1,^75^ corroborating a contribution of this region to thermal activation sensitivity in the fruit fly dTRPA1.^77^^,^^78^ In vertebrates, two regions of 6 ARs each in the snake thermosensitive TRPA1 (AR3-8; AR10-15) have been shown to revert the channel thermal sensitivity by conferring heat sensitivity to a chimeric AR hTRPA1.^75^ The temperature-dependent dynamics of the ARD have also recently been investigated in the TRPV1 channel, demonstrating that the ARD undergoes structural changes at similar temperatures that lead to TRPV1 activation, which suggested a potential role in the temperature-dependent structural changes leading to the channel opening.^77^ The N terminus region of mosquito TRPA1 also seems to be quite critical for heat sensitivity;^19^ however, there have been contradicting data for TRPA1, both from human and mosquito, arguing that additional regions controlling thermosensitivity are located outside the ARD.^26^^,^^79^^,^^80^ Altogether, with the understanding of limitations and constraints inherent to AlphaFold, these structural insights provide an interesting and relevant assessment of conserved key features of ankyrin TRPs for RpTRPA5B and underlie relevant structural novelties that may guide further functional studies in disentangling the proximate molecular determinants of the channel thermal and biophysical activation properties.

### Perspective considering plausible physiological roles for TRPA5 in thermosensation

Sensory receptors in the same clades are often tuned to detect a stimulus over a discrete window of intensities, enabling the recognition of physiologically relevant cues over a wide dynamic range.^1^^,^^3^^,^^81^ The TRP Ankyrin family is an excellent example of this pattern as distinct, yet closely related, channels account for thermal responsiveness over a range from innocuous to noxious heat.^4^^,^^12^^,^^82^ In addition, orthologous thermoTRPs often have different activation temperatures, and this has been postulated to reflect functional adaptive evolution to different optimal temperatures, coordinating thermoregulatory behaviors such as host seeking, thermal avoidance, and tracking of optimal temperatures.^83^

Despite living in a wide range of ecological conditions, insects show overall little variability in maximum temperature that they can tolerate in an active state without inducing neural and physiological damage (40°C–50°C),^84^ except certain thermophilic ants that can forage above 50°C for limited periods of time.^85^^,^^86^^,^^87^ In lab-simulated natural environments and in field thermal imaging studies, insects can reach 60°C under full sun with high humidity in as little as 15 s,^87^ corroborating early studies showing that the body temperature of insects heated by the sun is greater than the surrounding air temperature since insects are small physical bodies absorbing radiation.^88^^,^^89^ Considering that *Rhodnius*, and other triatomine insect adults in general, are about 3 cm in length and dark colored, with a small thermal capacity, and that the majority of species typically inhabit tropical areas with high humidity, their bodies could therefore rapidly reach high temperatures if exposed to full sun. Interestingly, the upper physiological thermal tolerance of *R. prolixus* and *T. infestans*, delineated by the maximal temperature at which these species can no longer respond to further changes in temperatures, leading notably to the onset of muscle spasms and respiratory breakdown, has been recently established at 50.5°C and 53.4°C, respectively.^90^ This suggests that the thermosensitivity established for RpTRPA5B may be in line with physiologically noxious environmental temperatures. The perception of environmental temperatures occurs through various organs and through the nervous system.^86^ RpTRPA5B is expressed broadly across tissues similarly to other insect thermoTRPs,^18^^,^^28^ which, together with functional validation of the channel *in vitro* activation by temperature, is in line with a possible physiological role in thermosensation. To investigate a possible role in physiological thermotolerance of TRPA5, it will be interesting to map the cellular location of TRPA5 in peripheral sensory neurons, including neural populations in the central nervous system, as shown in dTRPA1-D or Pyrexia channels,^18^^,^^22^ which are associated with physiological thermotolerance in the fruit fly.^86^

Variable environmental temperatures are extremely common in natural environments of small insects with low thermal capacity; thus, detecting and avoiding heat are critical to prevent injury. Temperature distributions also vary widely for natural objects. For instance, dry and moderately gray-colored or dark objects such as rocks or tree bark easily reach temperatures above 50°C.^87^ If the humidity level is high, and radiative cooling of the sky is not effective, the same objects can reach temperatures above 60°C in the full sun. Temperatures of dry leaf substrates on the ground can exceed 50°C in full sun since they do not undergo evaporative cooling, which would typically prevent a live leaf’s surface temperature from going above 40°C. Body temperatures of insects sampled at the surface of leaves in full sun tend to track leaf temperatures, although systematically above by up to 5°C–10°C.^89^ Other sunlight-exposed natural materials, such as sand or porous rock, have been observed to reach peak surface temperatures ranging between 61°C and 76°C under ambient temperatures of 48°C and solar irradiation 940 W/m^2^.^91^

Triatomines occur throughout widely diverse habitats, with variable diel activities, and can be associated with vertebrate fauna in, e.g., underground caves, palm trees, or rocky mounds throughout the Americas and are subjected to variable environmental temperatures.^92^ Evolutionarily, insects of the *Rhodnius* genus prevailed naturally in sylvatic environments, in the crowns of *Atalea* palm trees that typically experience a microclimate moderately oscillating between 24°C and 27°C,^93^ whereas colonization of environments associated with humans is a recent adaptation. Noxious activation temperatures like those reported for RpTRPA5B (>50°C) thus may appear outside of *Rhodnius* natural ecological temperature preferences. *R. prolixus* are also rarely seen in bright daylight due to strong negative phototaxis and intense thigmotaxis,^39^ although *Trypanosoma* infection can alter phototaxis and locomotor activity.^94^^,^^95^ Conversely, triatomines like *Triatoma infestans or T. brasiliensis* that inhabit rocky mounds in semi-arid environments, are intensely exposed to, and can be active in, plentiful sunlight^92^ with high ambient temperatures and sun heat-exposed surfaces.^91^ Many triatomine species, and by extension other insect groups, therefore offer a pertinent natural history^92^ and ground to be able to detect information from nociceptors capable of sensing high temperatures that they could exploit in a relevant behavioral context. Behavioral assays recording noxious thermotactic responses of mutant flour beetles *T. castaneum* and fruit flies have previously corroborated TRPA1 or painless TRP activation thresholds determined via heterologous expression.^17^^,^^96^^,^^97^ In addition, our *in vitro* assay reconciled the activation thresholds of dTRPA1-D and rTRPV1 with the known reported physiological threshold of perception for these channels.^22^^,^^64^ In this context, it will be intriguing to couple genetic (e.g., RNAi silencing^49^) and behavioral experiments targeting TRPA5 to determine how the established thermosensitivity of the channel reconciles with *in vivo* noxious behavioral responses linked to inner temperature regulation and heat substrate avoidance.

Finally, several classes of thermoTRPs have been established as polymodal sensors of physical and chemical stimuli.^81^ From a general evolutionary perspective, members of the TRPA5 clade may also function as polymodal sensors exhibiting heat and chemical sensitivity, although potential candidate chemical activators remain to be identified and tested both *in vitro* and *in vivo*. For instance, channels in the insect TRPA1 and HsTRPA clades are typically activated by allyl isothiocyanate (AITC) and various plant-derived chemicals such as carvacrol and citronellal.^23^^,^^28^^,^^29^ Reversely, characterized receptors of noxious heat in insects such as Pyrexia and Painless do not exhibit chemical sensitivity to electrophiles.^17^ *Rhodnius* is an insect model amenable to sensory behavioral manipulation. Recently, live *Rhodnius* individuals treated with capsaicin, the vanilloid pungent extract of chili peppers, were shown to have impaired orientation toward a thermal source,^98^ although which and if TRPs are involved remains to be determined. Notably, capsaicin can directly activate the mammalian TRPV1 receptor independent of temperature, and the mammalian noxious temperature receptor, TRPV2, when bearing only four mutations.^3^^,^^15^ Other than capsaicin, both mammalian TRPV1 and TRPV2 are readily activated by additional vanilloid compounds such as resiniferatoxin, an active compound from the cactus *Euphorbia resinifera* used for medicinal purposes and other plant-derived compounds that act as chemical agonists.^99^ Altogether, *in vivo* behavioral exposure of *Rhodnius* individuals combined with gene expression knockdown would also be interesting in search of botanical compounds potentially triggering TRPA5 activation in *Rhodnius*, *T. infestans* and hemipterans that share a close TRPA5B orthologue such as the bed bug, *Cimex lectularius*.

In conclusion, our study establishes the biophysical properties, and activation range of an ankyrin TRPA5 ion channel sensitive to a wide range of temperatures *in vitro*, which highlights independent evolutionary origins of the molecular transduction of thermal stimuli in insects. Our study opens the door for exploring the functional range of TRPA5 orthologues across triatomine vectors and other insects, and their physiological role for inner temperature regulation and noxious heat sensing.

### Limitations of the study

This study demonstrates the biophysical properties of a previously uncharacterized insect TRPA5 ion channel as a thermoTRP displaying thermosensitivity across a wide range of temperatures. However, behavioral functional experiments would be necessary to prove its role and importance in detecting noxious thermal stimuli.

## STAR★Methods

### Key resources table

**Table:** 

REAGENT or RESOURCE	SOURCE	IDENTIFIER
**Antibodies**		

Monoclonal anti-FLAG M2 antibody produced in mouse	Sigma-Aldrich	Cat#F1804; RRID:AB_262044
Sheep Anti-Mouse IgG - Horseradish Peroxidase	Cytiva	Cat#NA931; RRID:AB_772210
Rabbit Anti-Sodium Potassium ATPase monoclonal antibody, unconjugated, clone EP1845Y	Abcam	Cat#ab76020; RRID:AB_1310695

**Bacterial and virus strains**		

*Stbl3* competent *E. coli* cells	ThermoFisher	Cat#C737303

**Biological samples**		

*Rhodnius prolixus* antennae	Prof. Ian Orchard, University of Toronto Mississauga, Canada	RRID:NCBITaxon_13249
*Rhodnius prolixus* live adults	BEI	https://www.beiresources.org/Catalog/BEIVectors/NR-44077.aspx; RRID:NCBITaxon_13249

**Chemicals, peptides, and recombinant proteins**		

Lipofectamine 2000	Life Technologies	Cat#11668019
Opti-MEM I Reduced Serum	Life Technologies	Cat#31985070
Accutase	Stemcell Technologies	Cat#07920
DMEM High Glucose, GlutaMAX	Life Technologies	Cat#10566016
Fetal Serum Bovine, Seradigm Premier	VWR	Cat#97068-085

**Critical commercial assays**		

Direct-zol RNA kit	Zymo Research	Cat#R2051
GoScript™ Reverse Transcriptase kit	Promega	Cat#A5003
Advantage® 2 PCR Kit	Takara Bio	Cat#639206
Monarch® DNA gel extraction kit	NEB	Cat#T1020S
Qiaprep spin Miniprep	Qiagen	Cat#27104
endo-free ZymoPURE™ II Plasmid Midiprep Kit	Zymo Research	Cat#D4201
Pierce Cell surface Protein isolation kit	Thermo Scientific	Cat#89881
Trans-Blot Turbo RTA Mini 0.2 μm Nitrocellulose Transfer kit	Bio-Rad	Cat#1704270

**Deposited data**		

Sequence Read Archive (SRA) PRJNA281760/SRA:SRP057515 at NCBI	Latorre-estivalis et al.^45^	https://www.ncbi.nlm.nih.gov/sra/?term=SRP057515
Sequence Read Archive (SRA) PRJEB13049/SRA:ERP014587 at NCBI	NCBI	https://www.ncbi.nlm.nih.gov/sra/?term=ERP014587
Sequence Read Archive (SRA) PRJNA191820/SRA:SRP006783 at NCBI	NCBI	https://www.ncbi.nlm.nih.gov/sra/?term=SRP006783
*R. prolixus* genome assembly version RproC3.0.3	Mesquita et al.^36^	VectorBase.org
dTRPA1 structure state 1	Wang et al.^59^	PDB: 7YKR
dTRPA1 structure state 2	Wang et al.^59^	PDB: 7YKS
hTRPA1 structure	Zhao et al.^61^	PDB: 6V9Y

**Experimental models: Cell lines**		

Human: HEK293T	Sigma-Aldrich	Cat#12022001; RRID:CVCL_0063

**Oligonucleotides**		

Primers for TRP amplification, subcloning, sanger verification and quantitative PCR analyses	See Table S7	N/A

**Recombinant DNA**		

Plasmid DNA, rTRPV1 in pcDNA3	Prof. Rachelle Gaudet, MCB department, Harvard University	N/A
Plasmid DNA, dTRPA1-D in pcDNA3	Zhong et al.^22^	N/A
Expression vector pcDNA5-FLAG-T2A-mRuby2	Lienard et al.^51^	N/A
Plasmid DNA, RpTRPA5B in pcDNA5-FLAG-T2A-mRuby	This paper	N/A
Plasmid DNA, rTRPV1 in pcDNA5-FLAG-T2A-mRuby2	This paper	N/A
Plasmid DNA, dTRPA1-D in pcDNA5-FLAG-T2A-mRuby2	This paper	N/A

**Software and algorithms**		

NCBI-Blast	Altschul et al.^104^	https://blast.ncbi.nlm.nih.gov/Blast.cgi
MAFFT	Katoh and Standley^107^	https://mafft.cbrc.jp/alignment/server/index.html
IQ-Tree v1.6.11	Nguyen et al.^108^ Trifinopoulos et al.^109^ Kalyaanamoorthy et al.^110^	http://www.iqtree.org
R studio (R V3.6.3) ggtree package	Yu^111^	https://bioconductor.org/packages/release/bioc/html/ggtree.html
Evolview	Zhang et al.^112^	https://evolgenius.info/evolview-v2/
Cutadapt version 1.16	Martin^113^	https://cutadapt.readthedocs.io/en/v1.16/
HISAT2 version 2.2.0	Kim et al.^114^	http://daehwankimlab.github.io/hisat2/download/
StringTie version 2.1.3b	Pertea et al.^115^	https://ccb.jhu.edu/software/stringtie/
Trinotate version 3.2.1	Bryant et al.^116^	https://github.com/Trinotate/Trinotate/releases
Apollo gene browser	Lee et al.^117^	http://genomearchitect.readthedocs.io
R studio (R V3.6.3) tximport package	Soneson et al.^118^	https://bioconductor.org/packages/release/bioc/html/tximport.html
Geneious	Kearse et al.^119^	https://www.geneious.com
CFX Maestro Software 2.3 (Biorad)	N/A	https://www.bio-rad.com/en-be/category/qpcr-analysis-software?ID=42a6560b-3ad7-43e9-bb8d-6027371de67a
AlphaFold2	Jumper et al.^56^ Mirdita et al.^58^	https://alphafold.ebi.ac.uk
PyMOL	Pymol^120^	https://pymol.org/2/
DALI server	Holm^121^	http://ekhidna2.biocenter.helsinki.fi/dali/
Labview	Emerson	https://www.ni.com/en.html
Digidata 1440A ,pClamp10 software	Molecular Devices	https://info.molecularmolecul.com

**Other**		

DOI link for source data related to Figure 1	This paper	https://figshare.com/s/2d1ad932f34bdca2a769
DOI link for source data related to Figure 2	This paper	https://figshare.com/s/995be46d32030cd52910
DOI link for source data related to Figures 3 and S7–S10	This paper	https://figshare.com/s/0606a76fe5e96202ef73
DOI link for source data and code related to Figure S1	This paper	https://figshare.com/s/aca16873b4cc7e62d242
DOI link for source data and code related to Figure S2	This paper	https://figshare.com/s/014ec6a509c3c3d01896
DOI link for source data and code related to Figure S3	This paper	https://figshare.com/s/09aa5dfd07dc56ee1bcc
DOI link for source data related to Figure S4	This paper	https://figshare.com/s/85aa2e7bfcd7a3bc4168
DOI link for source data related to Figure S5	This paper	https://figshare.com/s/995be46d32030cd52910
DOI link for source data related to Figure S6	This paper	https://figshare.com/s/789896f4ab3b1804a648

### Resource availability

#### Lead contact

Further information and requests for resources and reagents should be directed to and will be fulfilled by the lead contact, Marjorie Liénard (Marjorie.lienard@biol.lu.se).

#### Materials availability

Plasmids generated in this study are available upon simple request to the lead contact.

#### Data and code availability

• Data S1 in the supplemental information lists the source data files associated with each main and SI figure. The DOI links are listed in Data S1 and the key resources table. Original western blot images are deposited in Figshare and are publicly available as of the date of publication.
• This paper analyses publicly available data. The accession numbers for the datasets are listed in the key resources table.
• All original code used to generate phylogenies and graphs has been deposited at Figshare and is publicly available as of the date of publication. DOI links are listed in the key resources table.
• Any additional information required to reanalyze the data reported in this paper is available from the lead contact upon request.

### Experimental model and study participant details

#### *Rhodnius prolixus*

Adults were obtained from BEI Resources (USA). Upon reception, animals were taken to a secure animal facility, asleep individually with carbon dioxide, and desired tissues dissected with RNase-free dissection tools, and pooled. Each tissue type was stored in DNA/RNA-free tubes filled with DNA/RNA shield reagent (Zymo) and stored at -20°C until further processing.

#### Cell line

Commercial HEK293T cells were purchased from Sigma-Aldrich and cultured in DMEM High Glucose, GlutaMAX (Life Technologies) supplemented with 10% FBS (Seradigm Premium, VWR, USA). Cells were incubated at 37°C in a humidified HERAcell 150i incubator (Thermo Scientific) with 5% CO_2_. The cell lines were tested negative for Mycoplasma.

#### Age and developmental stage of study participant or experimental model

Adult stages of *Rhodnius prolixus* insects were used.

### Method details

#### Phylogenetic analyses

Amino acid sequences of insect TRPA channels from the Anoplura (sometimes included under Psocodea or Phthiraptera), Coleoptera, Diptera, Hemiptera, Hymenoptera, Isoptera and Lepidoptera insect orders were retrieved from the InsectBase repository,^101^ FlyBase version FB2020_03,^102^ VectorBase (https://www.vectorbase.org), BeeBase,^103^ NCBI-blast,^104^ EnsemblMetazoa (https://metazoa.ensembl.org), the i5k Workspace@ NAL^105^ and OrthoFinder.^106^ The TRP sequences from insect model systems including *Drosophila melanogaster, Tribolium castaneum, Bombyx mori, Apis mellifera* and *Rhodnius prolixus* were used as templates to mine and curate orthologous TRP ORF sequences from annotated insect genomes and transcriptomes. To classify the uncharacterized TRPs, amino acid sequences were aligned using MAFFT,^107^ and Maximum-Likelihood phylogenetic trees were inferred in IQ-TREE v1.6.11 using ModelFinder (Ultrafast Bootstrap, 1000 replicates), using a best-fit model JTT+F+I+G4 measured by the Bayesian information criterion (BIC).^108^^,^^109^^,^^110^ The phylogenetic trees were visualized, rooted at mid-point and annotated in R V3.6.3 using the ggtree package^111^ and Evolview.^112^ The accession numbers are listed in Table S1.

#### TRPA5 gene annotation and expression

We collected Illumina read data from *R. prolixus* tissue libraries published in the Sequence Read Archive (SRA) at NCBI under Bioproject accession numbers PRJNA281760/SRA:SRP057515 (antennal library from larvae, female adult and male adult),^45^ PRJEB13049/SRA:ERP014587 (head library), and PRJNA191820/SRA:SRP006783 (ovary and testes library). We performed low-quality base trimming and adaptor removal using cutadapt version 1.16^113^ and aligned the trimmed read pairs against the *R. prolixus* assembly version RproC3.0.3 (retrieved from VectorBase.org) genome using HISAT2 version 2.2.0.^114^ The existing annotation was used to create a list of known splice sites using a python script distributed with HISAT2. We used StringTie version 2.1.3b^115^ with the *conservative* transcript assembly setting to improve the annotation, reconstruct a non-redundant set of transcripts observed in any of the RNA-Seq samples, and compute expression estimates.

We applied Trinotate version 3.2.1^116^ to generate a functional annotation of the transcriptome data. In particular, the functional annotation of TRP genes for which the initial genome annotation was absent or incomplete (i.e *TRPA5, Nan, Pain*) were localized in Trinotate annotation followed by validation using the Apollo gene browser.^117^ All TRP gene identifiers are presented in Table S2.

The alignment BAM files were used to estimate transcript abundance using StringTie together with our improved annotation. The abundance tables from StringTie were imported into R using the *tximport* package,^118^ which was used to compute gene-level abundance estimates reported as FPKM. We used the R package *pheatmap* to visualize the expression level of TRP genes.

#### Monitoring of TRPA5B expression levels

Female antennae, rostrum, legs, heads (minus antenna and rostrum), and bodies (thorax minus legs + abdomen) were dissected and pooled from 15 individuals in DNA/RNA shield reagent (Zymo) and stored at -20°C until further processing. Total RNA was isolated using the Monarch RNA extraction procedure (New England Biolabs), including tissue grinding in liquid nitrogen and a DNAse I step. cDNAs were synthesized using the GoScript cDNA synthesis procedure (Promega) prior to concentration assessment using the Qubit High sensitivity DNA kit (Invitrogen). Two gene-specific primer (GSP) sets were designed for *Rhodnius* Actin (Genbank:EU233794.1) and TRPA5B using Primer3 version 2.3.7 in Geneious^119^ (Table S5). Each primer set was initially validated by calculating standard curves from serial dilutions of template cDNA (2 ng/μL to 0.25 ng/μL) and primer mix (5 to 0.25 μM) with choosing amplification efficiencies (E) between 95 and 100%. qPCR amplification products from initial runs were additionally checked on 2% agarose gels to verify the correct amplicon sizes and the absence of primer dimers. As a final validation, qPCR products were purified using Exo-SAP (Fermentas) prior to Sanger sequencing to ensure product amplification specificity. Quantitative PCR reactions were then run in three technical replicates on a CFX384 Real-Time PCR system (Bio-Rad) with quantification and dissociation curves analyses performed for three independent experiments using the CFX Maestro Software 2.3 (Bio-Rad). Each five-microliter reaction contained 2.5 μL 2x SsoAdvanced Universal SYBR Green Supermix (Biorad), 0.25 ng cDNA and 0.125 μM primers. Cycling conditions were as follows: 95°C for 2 min, 39 cycles of 95°C for 10 s, 60°C for 10 s followed by a dissociation curve analysis from 65.5°C to 89.5°C with gradual heating at 0.6°C/s. Relative log-fold expression levels were normalized per tissue type against the reference gene and calibrated relative to Antennae (log fold expression = 1).

#### AlphaFold modeling and DALI analyses

Monomer structures of *Rhodnius* TRPA1, *Rhodnius* Painless, *Rhodnius* Waterwitch, *Rhodnius* TRPA5B, *Drosophila* TRPA1-D, *Drosophila* Painless, *Drosophila* Waterwitch and *Drosophila* Pyrexia were generated using AlphaFold2 with amber relaxation activated^56^ on Colab’s server.^58^ The sequence of dTRPA1 isoform D used for the AlphaFold prediction is identical to the sequence of the determined structure (isoform A) except for the first 97 amino acid residues, in which the five last residues correspond to the first five in the resolved N-terminal region in 7YKR. Using the same tool, a tetrameric model of residues 477-1153 of *Drosophila* TRPA1-D corresponding to PDB ID 7YKS was made. No template was used in these predictions. To model the Rhodnius TRPA5B tetramer, due to limitations in computational power, the transmembrane region (residues 608-1078) was modelled first, and then used as a custom template to model a monomer of residues 42-1078. The first 41 residues and the C-terminal of the monomers from residue 1079 were disordered and truncated to avoid clashes when assembling the tetramer. A tetramer was assembled of four copies of the monomer by aligning them to each of the chains of the truncated transmembrane tetramer in PyMOL.^120^ The monomer models were compared with pairwise structural alignment using the Dali server.^121^ The PDB files are provided as source datafiles (Key resources table).

#### Molecular cloning

Antennae from twenty Rhodnius adult individuals were obtained from a laboratory culture (Orchard lab, University of Toronto Mississauga, Canada) and stored in DNA/RNA Shield™ reagent (Zymo Research). Tissues were disrupted in Trizol using a Premium Multi-Gen 7XL Homogenizer (PRO Scientific) and RNA was subsequently extracted using the Direct-zol RNA kit (Zymo Research), including a DNAse step to remove genomic DNA contamination. cDNA was synthesized from 1ug Total RNA using the GoScript™ Reverse Transcriptase kit (Promega) and random hexamers following the recommended manufacturers’ protocol. RNA and cDNA qualities were verified using a Nanodrop (Nanodrop 2000/2000c UV-vis spectrophotometer, Thermo Scientific) and quantified using a Qubit Fluorometer (ThermoFisher). The coding regions of Rhodnius *Rp-TRPA5B* was amplified from antennal cDNA using gene-specific primers designed based on Rhodnius full length TRP sequences^36^ and containing unique restriction sites (Table S6). PCR reactions were performed in a Veriti™ Thermal Cycler (ThermoFisher) using the Advantage® 2 PCR Kit (Takara Bio) in a touchdown cycling program as follows: 95°C for 2 min, 16 cycles of 95°C for 30 sec, 68°C for 1 min (-0.5°C/cycle), 68°C for 4 min followed by 20 cycles of 95°C for 30 sec, 60°C for 1 min, 68°C for 4 min, and a final step at 68°C for 10 min. Amplification products were analysed by electrophoresis, and fragments of expected size were excised from the gel, purified using the Monarch® DNA gel extraction kit (NEB) and subjected to Sanger Sequencing for ORF sequence-verification prior to codon-optimization at Genscript and subcloning. For the rat rTRPV1 and the fruit fly dTRPA1-D, gene specific primers (Table S6) were used to amplify the ORF including suitable flanking restriction sites prior to gel purification and double restriction digestion. The digested PCR products were gel purified and ligated in an expression cassette containing the human cytomegalovirus (CMV) immediate early promoter and engineered to include a C-terminal tag by the monoclonal antibody FLAG epitope sequence (DYKDDDDK), followed by a Ser-Gly-Ser linker peptide, a T2A peptide sequence (EGRGSLLTCGDVEENPG) and the coding region of the cytoplasmic fluorescent marker protein mRuby2.^51^^,^^100^ The ligation mixtures were used to transform *Stbl3* competent *E. coli* cells (ThermoFisher) using standard protocols. Plasmid DNAs were purified using the Qiaprep spin Miniprep (Qiagen) and verified by Sanger sequencing using internal gene-specific and vector primers to ensure overlapping sequence information in both forward and reverse directions. High yield pure plasmid DNA preparations were subsequently obtained from 100 mL overnight LB broth cultures using the endo-free ZymoPURE™ II Plasmid Midiprep Kit (Zymo Research, USA).

#### Transient HEK293T cell expression

Plasmid DNAs clones from TRP cDNAs were transiently expressed in HEK293T cells to optimize expression conditions via mRuby2 visualization and western blot analysis prior to whole cell patch clamp recordings. HEK293T cells were seeded at a density of 0.6 x 10^6^ cells on day 0 in 60 mm culture dishes (ref 25382-100, VWR). For each transfection, lipid complexes containing 2.5 μg DNA: 10 μL Lipofectamine 2000 (Life Technologies) mixed in Opti-MEM I Reduced Serum (Life Technologies) were added dropwise to the cells at 50% confluency (1.2 x 10^6^ cells). The culture medium was exchanged with new DMEM/FBS medium six-hours post-transfection.

#### Biochemistry

For whole-cell TRP expression analysis, cells were harvested 72h post-transfection; the medium was decanted, cells were collected in 2mL cold D-PBS, centrifuged for 5 min at 4,000 rpm at 4°C and then the supernatant was discarded. The cell pellet was gently suspended in 50 μL cold Ripa lysis buffer (Thermo Scientific) supplemented with 1% Triton-X100 (Sigma-Aldrich) and complete EDTA-free protein inhibitors (Sigma-Aldrich). Cell membranes were lysed for 1h at 4°C with gentle rotation on a sample homogenizer, and cell debris were collected by centrifugation at 4°C for 15 min at 13,000 rpm. The crude protein lysate concentration was quantified by bovine serum albumin (BSA) (Sigma-Aldrich) and 25 μg crude extract was loaded on NuPAGE™ 3-8% Tris-Acetate gels (ThermoFisher) and transferred to a polyvinylidene difluoride membrane on a TurboBlotTransfer system (Bio-Rad Laboratories). The membranes were blocked with 5% milk (Bio-Rad) in Tris-buffered saline containing 0.1% Tween 20 (TBST, Bio-Rad) and incubated overnight with aFLAG antibody 1:2,500 (GE Healthcare) on a gently rocking platform at 4°C. After washing with TBST the membranes were incubated for 1h at ambient temperature in the dark with horseradish peroxidase (HRP) ECL anti-mouse conjugated antibody (Amersham, USA) diluted in 5% milk in TBS-Tween at 1:2,500. Membranes were rinsed in TBST and revealed using the SuperSignal West Femto (Thermo Scientific) and imaged on a ChemiDoc system (Bio-Rad Laboratories).

For membrane surface expression, the plasma membrane expression of RpTRPA5B channels was assessed using the Pierce Cell surface Protein isolation kit (Thermo Scientific). On day 0, four T75 cm^2^ flasks were seeded with 1 x 10^6^ HEK293T cells and incubated at 37°C. Forty hours later, each flask was transfected with lipid complexes containing 48 μg endo-free plasmid DNA and 96 μl Lipofectamine 2000 diluted in Opti-MEM serum and incubated at 30°C. 72 hours post-transfection, cells were gently washed with ice-cold PBS, labeled with Sulfo-NHS-SS-Biotin, and harvested following the manufacturer’s protocol. Cells were lysed on ice for 30 min in the manufacturer’s lysis buffer supplemented with 0.5% Triton-X100 and complete EDTA-free protein inhibitors (Sigma-Aldrich), with gentle 5s vortexing every 5 min, and two 5x-1s sonicating bursts on ice. Following centrifugation, the cell lysate was bound to NeutrAvidin agarose resin and gently mixed for 60 min at ambient temperature on a platform rotator. The membrane-bound fraction was eluted with 50mM Dithiothreitol in SDS-Sample buffer (62.5 mM Tris/HCl pH6.8, 1% SDS, 10% Glycerol) and then placed on ice. For Western Blot analysis, 50 μg of the membrane protein eluate fraction quantified by BSA were diluted to 32 μl in lysis buffer containing loading Laemmli buffer (Bio-Rad) supplemented with 10% 2-mercaptoethanol. Sixteen μl (25 μg) of the homogenized protein-loading buffer sample were loaded in duplicates on a NuPAGE™ 3-8% Tris-Acetate gel (ThermoFisher) to be probed separately with FLAG and ATPase antibodies. Proteins were separated by electrophoresis for 3h at 80V at 4°C, then transferred to a polyvinylidene difluoride membrane on a TurboBlotTransfer system (Bio-Rad Laboratories). The membranes were blocked in parallel with 5% milk (Bio-Rad) in Tris-buffered saline containing 0.1% Tween 20 (TBS-T, Bio-Rad) and incubated overnight on a gently rocking platform at 4°C with aFLAG antibody 1:2,500 (Sigma-Aldrich) or with Anti-Sodium Potassium ATPase antibody 1:2,500 (Abcam) diluted in 5% milk. After three washes with TBST, the membranes were incubated for 1h at ambient temperature in the dark with HRP ECL anti-mouse conjugated antibody (Amersham, USA) at a 1:2,500 dilution in 5% milk/TBST. Membranes were rinsed in TBST and revealed using the SuperSignal West Femto (Thermo Scientific) and imaged on a ChemiDoc system (Bio-Rad Laboratories).

#### Temperature control using a laser system

We used a manual patch-clamp station (Axopatch 200, Molecular Devices) equipped with a fiber-delivered laser system to record temperature-activated currents under a precise voltage-clamp control. The setup was modified after Yao et al. (2009)^122^ and takes advantage of water’s IR absorption band to generate rapid temperature jumps from RT to high temperatures. It combines an infrared diode laser (λc =1460 nm (+/-20 nm), Output power = 4.8 watts) (Seminex Inc.) coupled with a 100-um optical fiber with a striped tip (ThorLabs, Inc.) as the controllable heat source. Two independent micromanipulators allowed us to precisely align the relative positions of the patch-clamp electrode and the fiber on a single cell (Figures 3 and S6). To calibrate the optic fiber position with respect to the patch pipette, we switched the laser system to a visible laser (red, λ = 633nm) (Figure S6) for visualizing the position of laser under the microscope. Marks on the computer screen were used to keep the position of the fiber and the pipette consistent for the different experiments. Cells under whole-cell voltage-clamp control were held at -30mV during the experiment. To program fast pseudo-transient temperature changes, the patch pipette current was used to read the temperature changes in real-time as the feedback to the laser diode controller (LDC-37620, ILX Lightwave) to perform proportional-integral-derivative (PID) control of the driving current of the laser diode. This laser-heating setup provides a rapid and precise heating rate on the order of 50°C within tens of milliseconds, essential to provide both adequate temporal resolution and controllable steady-state temperatures in the range of 35°C to 70°C to analyze the channel activation. Figure S9A shows constant temperature steps were achieved with a rising time constant of 34.2±3.3 ms, independent of the laser power. The temperature jump associated with successive current pulses is precisely calculated by running an open pipette calibration following the same current sequence at the end of each run (Figure 3D).

#### Temperature calibration

We used the resistance of the open pipette to measure the temperature jump magnitudes following the equation T={1/T_0_ – R/E_a_ x ln(I/I_0_)} - 1, where R is the gas constant, T_0_ and I_0_ are respectively room temperature and the corresponding electrode current at room temperature. The activation energy (E_a_) of the system corresponds to 3.84 kcal/mol as was established by Yao et al. (2009) for the pair of solutions used in the recordings.^122^ The equation describes the change in ion motility as a function of temperature changes in the system. The current change was used as a feedback signal for a laser-diode controller software coded in Labview that uses a proportional-integral-derivative (PID) control algorithm. To account for the variability in the diameter between the different patch pipettes used in different experiments, the instrument was calibrated before each experiment to assure comparable temperature jumps in each experiment, adjusting the diode power outputs to the desired temperature accordingly.

#### PID control

A PID (proportional-integral-derivative) control is the typical way to adjust the output according to the input reading in real-time without knowing most of the environmental parameters. The idea is described in the following function:

$$Output\left(t\right)=K_{p}Err\left(t\right)+K_{i}\int_{0}^{t}Err\left(\tau \right)d\tau +K_{d}\frac{dErr\left(t\right)}{dt}$$

Err(t)=Input(t)−Setpoint

with Output(t) being the laser power, Input(t) being the temperature, and Setpoint being the desired value. The Output(t) is determined by Err(t), which is the difference between Input(t) and Setpoint.

There are 3 terms in this equation, the first term is the proportional term. This term varies linearly with Err(t). For instance, when the temperature reaches the setpoint, this term decreases, and when the temperature exceeds the setpoint, this term becomes negative to bring the temperature back to the setpoint. The second term is the integral term. This term provides a gradually increasing offset and this offset will stabilize when the temperature stabilizes to setpoint, where Err(t)→0. The third term is the derivative term. This term estimates the required change of output by watching the inertia of Err(t). For instance, when the temperature reaches the setpoint, the proportional term gives 0 and the integral term gives a stabilized value, but if the temperature is still increasing, this term will decrease the output to prevent the temperature from exceeding the setpoint in the next time interval.

#### Open pipette current measurements

The temperature is measured by monitoring the current through an open patch pipette, which means there are no cells but only water, assuming the thermal property of the cell is the same as water. First, a series of open-pipette measurements with different laser powers and power waveforms is performed and used to calculate the temperature evolution from real-time current in the patch-clamp recording pipette. The patch-clamp experiment is conducted by applying the same laser powers and waveforms to the cell.

#### Fiber preparation

The attenuation coefficient of water at the wavelength of 1460 nm is ∼3000 *cm*ˆ(−1) which means the absorption of the laser power through a 100*nm* thick water layer is over 40%. If the laser is delivered from above the water surface,most of its optical power would thus be absorbed in the upper water layer. The temperature of water above the targeted cell would be much higher than the cell temperature itself, and the temperature change would not be confined to a single cell due to conduction and convection in the water. To resolve this, we cleaved the fiber with a ruby blade under a stereomicroscope and stripped the fiber which allowed us to place the fiber tip in the water layer directly above the cell in the recording chamber (Figure S6). Therefore we can precisely confine the area to be heated up. The relative position of the fiber tip and cell was adjusted using patch-clamp rig microscope.

#### Whole cell patch-clamp recordings

Cells were seeded at low density in a 30 mm culture dish (VWR) containing round glass coverslips 48h post-transfection (Table S7; Figure 3). Cells were first rinsed with D-PBS at room temperature, trypsinized with 0.5 mL Accutase (Stemcell Technologies) and suspended in 4.5 mL pre-warmed DMEM-FBS medium. Two hundred microliters of this cell suspension were mixed with 1.8 mL pre-warmed DMEM-FBS medium, dispensed drop wise in the culture dish, and incubated for 24h at 30°C. In a typical experiment, one glass cover slip was gently retrieved from the culture dish using sterile forceps, rinsed with a recording solution using a Pasteur pipette and placed in the recording chamber. The fluorescence of mRuby-expressing cells was monitored to select bright, healthy, isolated cells for whole-cell patch clamping. Experiments under the whole-cell configuration were carried out 72h after induction. The electrodes were fabricated with borosilicate capillaries, using a horizontal micropipette puller P-1000 (Sutter Instrument, Novato, CA, USA), and polished using a microforge (Narishige, Japan) to a final diameter between 2-4 um. The internal electrode was filled with the following solution in mM: 126 CsCl, 10 EGTA, 10 HEPES, 1 EDTA, and 4 MgATP. The extracellular recording solution contained 2 mM CaCl_2_, 10 mM HEPES, 140 mM NaCl, pH 7.2 (adjusted with NaOH). The electrode resistance ranged between 2-4 MΩ, and the Vjp was estimated at ∼18 mV for the recordings. The current traces were amplified using a MultiClamp 700B amplifier (Molecular Devices, Sunnyvale, CA, USA). The amplified electrical signals were acquired with a Digidata 1440A using the pClamp10 software (Molecular Devices, Sunnyvale, CA, USA). Series Resistance (Rs) was compensated in 80%, as well as the fast and slow capacitive components of the current. The current density was fitted to the following Boltzmann function:

$$I\_total=I\_leak\;e^{\left(-\left(\Delta H\_leak\right)/RT\right)}+(I\_max\;e^{\left(-\left(\Delta H\_i\right)/RT\right))}/\left(1+e^{\left(-\left(\Delta G\_T\right)/RT\right)}\;\right)$$

Whereby the first term ΔH_leak is the enthalpy change of the leak current. The second term accounts for the channel activity, with ΔG=ΔH−TΔS is the free energy change involved in the closed-open reaction, and ΔH_i accounts for the linear temperature dependence of the ionic conductivity and leakage current.^122^ The corrected temperature current density (*I*) was used to calculate the equilibrium constant from the relative fraction of the channel in the open conformation (Po), assuming a two-state model, where Po = I/Imax.

$$Po=1/\left(1+Keq^{-1}\right)$$

where $\text{In}({\text{K}}_{\text{eq}})=-(\Delta \text{H}/\text{RT})+(\Delta \text{S}/\text{R})$. Thus from the van’t Hoff plots ln(Keq) vs 1/T, the enthalpy and entropy associated with the channel opening can be obtained.^72^ The channel temperature activation parameters are obtained from the normalized current (Gmax or I/Imax) following methods for both cold-and heat-activated ion channels.^52^^,^^70^ Normalization of the maximum amplitude against an agonist does not influence the thermodynamic parameters as determined from the normalized current.

## Supplemental information

Document S1. Figures S1–S10, Tables S3–S7, and Data S1

Table S1. TRPA gene accession numbers, related to Figures 1A, S1, and S2

Table S2. *Rhodnius prolixus* TRP gene identifiers, related to Figure 1B

## References

[1] McKemy D.D. (2007). Temperature sensing across species. Pflugers Arch..

[2] Vriens J., Nilius B., Voets T. (2014). Peripheral thermosensation in mammals. Nat. Rev. Neurosci..

[3] Xiao R., Xu X.Z.S. (2021). Temperature sensation: from molecular themosensors to neural circuits and coding principles. Annu. Rev. Physiol..

[4] Castillo K., Diaz-Franulic I., Canan J., Gonzalez-Nilo F., Latorre R. (2018). Thermally activated TRP channels: molecular sensors for temperature detection. Phys. Biol..

[5] Garrity P.A., Goodman M.B., Samuel A.D., Sengupta P. (2010). Running hot and cold: behavioral strategies, neural circuits, and the molecular machinery for thermotaxis in *C. elegans* and *Drosophila*. Genes Dev..

[6] Klein M., Afonso B., Vonner A.J., Hernandez-Nunez L., Berck M., Tabone C.J., Kane E.A., Pieribone V.A., Nitabach M.N., Cardona A. (2015). Sensory Determinants of Behavioral Dynamics in Drosophila Thermotaxis. Proc. Natl. Acad. Sci. USA.

[7] Clapham D.E. (2003). TRP channels as cellular sensors. Nature.

[8] Venkatachalam K., Montell C. (2007). TRP channels. Annu. Rev. Biochem..

[9] Baez-Nieto D., Castillo J., Dragicevic C., Alvarez O., Latorre R. (2011). Thermo-TRP Channels: Biophysics of Polymodal Receptors. Adv. Exp. Med. Biol..

[10] Fowler M.A., Montell C. (2013). Drosophila TRP channels and animal behavior. Life Sci..

[11] Peng G., Shi X., Kadowaki T. (2015). Evolution of TRP channels inferred by their classification in diverse animal species. Mol. Phylogenet. Evol..

[12] Matsuura H., Sokabe T., Kohno K., Tominaga M., Kadowaki T. (2009). Evolutionary conservation and changes in insect TRP channels. BMC Evol. Biol..

[13] Caterina M.J., Schumacher M.A., Tominaga M., Rosen T.A., Levine J.D., Julius D. (1997). The capsaicin receptor: a heat-activated ion channel in the pain pathway. Nature.

[14] Gracheva E.O., JF C.-M., Carcacia-González J.A., Ingolia N., Manno C., Aranguren C., Weissman J., Julius D. (2011). Ganglion-specific Splicing of TRPV1 Underlies Infrared Sensation in Vampire Bats. Nature.

[15] Caterina M.J., Rosen T.A., Tominaga M., Brake A.J., Julius D. (1999). A capsaicin-receptor homologue with a high threshold for noxious heat. Nature.

[16] Vriens J., Owsianik G., Hofmann T., Philipp S.E., Stab J., Chen X., Benoit M., Xue F., Janssens A., Kerselaers S. (2011). TRPM3 Is a Nociceptor Channel Involved in the Detection of Noxious Heat. Neuron.

[17] Tracey W.D., Wilson R.I., Laurent G., Benzer S. (2003). painless, a Drosophila Gene Essential for Nociception. Cell.

[18] Lee Y., Lee Y., Lee J., Bang S., Hyun S., Kang J., Hong S.-T., Bae E., Kaang B.-K., Kim J. (2005). Pyrexia is a new thermal transient receptor potential channel endowing tolerance to high temperatures in Drosophila melanogaster. Nat. Genet..

[19] Kang K., Panzano V.C., Chang E.C., Ni L., Dainis A.M., Jenkins A.M., Regna K., Muskavitch M.A.T., Garrity P.A. (2011). Modulation of TRPA1 thermal sensitivity enables sensory discrimination in Drosophila. Nature.

[20] Liu L., Li Y., Wang R., Yin C., Dong Q., Hing H., Kim C., Welsh M.J. (2007). Drosophila hygrosensation requires the TRP channels water witch and nanchung. Nature.

[21] Hamada F.N., Rosenzweig M., Kang K., Pulver S.R., Ghezzi A., Jegla T.J., Garrity P.A. (2008). An internal thermal sensor controlling temperature preference in Drosophila. Nature.

[22] Zhong L., Bellemer A., Yan H., Ken H., Jessica R., Hwang R.Y., Pitt G.S., Tracey W.D. (2012). Thermosensory and Nonthermosensory Isoforms of *Drosophila melanogaster*TRPA1 Reveal Heat-Sensor Domains of a ThermoTRP Channel. Cell Rep..

[23] Guntur A.R., Gu P., Takle K., Chen J., Xiang Y., Yang C.-H. (2015). Drosophila TRPA1 isoforms detect UV light via photochemical production of H2O2. Proc. Natl. Acad. Sci. USA.

[24] Gu P., Gong J., Shang Y., Wang F., Ruppell K.T., Ma Z., Sheehan A.E., Freeman M.R., Xiang Y. (2019). Polymodal Nociception in Drosophila Requires Alternative Splicing of TrpA1. Curr. Biol..

[25] Wang G., Qiu Y.T., Lu T., Kwon H.-W., Pitts R.J., Van Loon J.J.A., Takken W., Zwiebel L.J. (2009). *Anopheles gambiae* TRPA1 is a heat-activated channel expressed in thermosensitive sensilla of female antennae. Eur. J. Neurosci..

[26] Survery S., Moparthi L., Kjellbom P., Hogestatt E., Zygmunt P.M., Johanson U. (2016). The N-Terminal Ankyrin Repeat Domain Is Not Required for Electrophile and Heat Activation of the Purified Mosquito TRPA1 Receptor. J Biol Chem..

[27] Sato A., Sokabe T., Kashio M., Yasukochi Y., Tominaga M., Shiomi K. (2014). Embryonic thermosensitive TRPA1 determines transgenerational diapause phenotype of the silkworm, Bombyx mori. Proc. Natl. Acad. Sci. USA.

[28] Kohno K., Sokabe T., Tominaga M., Kadowaki T. (2010). Honey Bee Thermal/Chemical Sensor, AmHsTRPA, Reveals Neofunctionalization and Loss of Transient Receptor Potential Channel Genes. J. Neurosci..

[29] Wang X., Li T., Kashio M., Xu Y., Tominaga M., Kadowaki T. (2018). HsTRPA of the Red Imported Fire Ant, *Solenopsis invicta*, Functions as a Nocisensor and Uncovers the Evolutionary Plasticity of HsTRPA Channels. eNeuro.

[30] Wigglesworth V. (1939).

[31] Guarneri A., Lorenzo M. (2021). Springer International Publishing.

[32] Coura J.R., Viñas P.A. (2010). Chagas disease: a new worldwide challenge. Nature.

[33] Clayton J. (2010). Chagas disease 101. Nature.

[34] Pérez-Molina J.A., Molina I. (2018). Chagas disease. Lancet.

[35] (2015). Chagas disease in Latin America: an epidemiological update based on 2010 estimates. Wkly Epidemiol Rec..

[36] Mesquita R.D., Vionette-Amaral R.J., Lowenberger C., Rivera-Pomar R., Monteiro F.A., Minx P., Spieth J., Carvalho A.B., Panzera F., Lawson D. (2015). Genome of Rhodnius prolixus, an Insect Vector of Chagas Disease, Reveals Unique Adaptations to Hematophagy and Parasite Infection. Proc. Natl. Acad. Sci. USA.

[37] Barrozo R.B., Reisenman C.E., Guerenstein P., Lazzari C.R., Lorenzo M.G. (2017). An inside look at the sensory biology of triatomines. J. Insect Physiol..

[38] Barrozo R.B., Lorenzo M.G., Guarneri A., Lorenzo M. (2021). Triatominae - The Biology of Chagas Disease Vectors.

[39] Lazzari C.R., Guarneri A., Lorenzo M. (2021). Triatominae - The Biology of Chagas Disease Vectors.

[40] Lazzari C.R., Núñez J. (1989). The response to radiant heat and the estimation of the temperature of distant sources in *Triatoma infestans*. J. Insect Physiol..

[41] Lazzari C.R., Nunez J.A. (1989). Blood temperature and feeding-behavior in *Triatoma infestans* (heteroptera: Reduviidae). Entomol. Gen..

[42] Guerenstein P.G., Lazzari C.R. (2009). Host-seeking: How triatomines acquire and make use of information to find blood. Acta Trop..

[43] Zopf L.M., Lazzari C.R., Tichy H. (2014). Differential effects of ambient temperature on warm cell responses to infrared radiation in the bloodsucking bug Rhodnius prolixus. J. Neurophysiol..

[44] Zopf L.M., Lazzari C.R., Tichy H. (2014). Infrared detection without specialized infrared receptors in the bloodsucking bug *Rhodnius prolixus*. J. Neurophysiol..

[45] Latorre-Estivalis J.M., Robertson H.M., Walden K.K.O., Ruiz J., Gonçalves L.O., Guarneri A.A., Lorenzo M.G. (2017). The molecular sensory machinery of a Chagas disease vector: expression changes through imaginal moult and sexually dimorphic features. Sci. Rep..

[46] Latorre-Estivalis J.M., Lorenzo M.G. (2019). Molecular bases of sensory processes in kissing bugs, vectors of Chagas disease. Curr. Opin. Insect Sci..

[47] Latorre-Estivalis J.M., Sterkel M., Ons S., Lorenzo M.G. (2020). Transcriptomics supports local sensory regulation in the antenna of the kissing-bug Rhodnius prolixus. BMC Genom..

[48] Chaverra-Rodriguez D., Macias V.M., Hughes G.L., Pujhari S., Suzuki Y., Peterson D.R., Kim D., McKeand S., Rasgon J.L. (2018). Targeted delivery of CRISPR-Cas9 ribonucleoprotein into arthropod ovaries for heritable germline gene editing. Nat. Commun..

[49] Pereira J., Diogo C., Fonseca A., Bomfim L., Cardoso P., Santos A., Dittz U., Miranda K., de Souza W., Gioda A. (2020). Silencing of RpATG8 impairs the biogenesis of maternal autophagosomes in vitellogenic oocytes, but does not interrupt follicular atresia in the insect vector *Rhodnius prolixus*. PLoS Negl. Trop. Dis..

[50] Latorre Estivalis J.M., Traverso L., Pontes G., Lorenzo M.G. (2022). The antennal transcriptome of Triatoma infestans reveals substantial expression changes triggered by a blood meal. BMC Genom..

[51] Liénard M.A., Bernard G.D., Allen A., Lassance J.M., Song S., Childers R.R., Yu N., Ye D., Stephenson A., Valencia-Montoya W.A. (2021). The evolution of red colour vision is linked to coordinated rhodopsin tuning in lycaenid butterflies. Proc. Natl. Acad. Sci. USA.

[52] Yao J., Liu B., Qin F. (2010). Kinetic and Energetic Analysis of Thermally Activated TRPV1 Channels. Biophys. J..

[53] Liu B., Qin F. (2017). Single-residue molecular switch for high-temperature dependence of vanilloid receptor TRPV3. Proc. Natl. Acad. Sci. USA.

[54] Tominaga M., Caterina M.J., Malmberg A.B., Rosen T.A., Gilbert H., Skinner K., Raumann B.E., Basbaum A.I., Julius D. (1998). The cloned capsaicin receptor integrates multiple pain-producing stimuli. Neuron.

[55] Liu B., Hui K., Qin F. (2003). Thermodynamics of heat activation of single capsaicin ion channels VR1. Biophys. J..

[56] Jumper J., Evans R., Pritzel A., Green T., Figurnov M., Ronneberger O., Tunyasuvunakool K., Bates R., Žídek A., Potapenko A. (2021). Highly accurate protein structure prediction with AlphaFold. Nature.

[57] Varadi M., Anyango S., Deshpande M., Nair S., Natassia C., Yordanova G., Yuan D., Stroe O., Wood G., Laydon A. (2022). AlphaFold Protein Structure Database: massively expanding the structural coverage of protein-sequence space with high-accuracy models. Nucleic Acids Res..

[58] Mirdita M., SchÜtze K., Moriwaki Y., Heo L., Ovchinnikov S., Steinegger M. (2022). ColabFold: making protein folding accessible to all. Nat. Methods.

[59] Wang X., Li Y., Wei H., Yang Z., Luo R., Gao Y., Zhang W., Liu X., Sun L. (2023). Molecular architecture and gating mechanisms of the Drosophila TRPA1 channel. Cell Discov..

[60] Paulsen C.E., Armache J.P., Gao Y., Cheng Y., Julius D. (2015). Structure of the TRPA1 ion channel suggests regulatory mechanisms. Nature.

[61] Zhao J., Lin King J.V., Paulsen C.E., Cheng Y., Julius D. (2020). Irritant-evoked activation and calcium modulation of the TRPA1 receptor. Nature.

[62] Cattaneo A.M., Bengtsson J.M., Montagné N., Jacquin-Joly E., Rota-Stabelli O., Salvagnin U., Bassoli A., Witzgall P., Anfora G. (2016). TRPA5, an ankyrin subfamily insect TRP channel, is expressed in antennae of *Cydia pomonella* (Lepidoptera: Tortricidae) in multiple splice variants. J. Insect Sci..

[63] Neely G.G., Keene A.C., Duchek P., Chang E.C., Wang Q.-P., Aksoy Y.A., Rosenzweig M., Costigan M., Woolf C.J., Garrity P.A., Penninger J.M. (2011). TrpA1 Regulates Thermal Nociception in Drosophila. PLoS One.

[64] Rosenzweig M., Brennan K.M., Tayler T.D., Phelps P.O., Patapoutian A., Garrity P.A. (2005). The Drosophila ortholog of vertebrate TRPA1 regulates thermotaxis. Genes Dev..

[65] Cheng W., Yang F., Liu S., Colton C.K., Wang C., Cui Y., Cao X., Zhu M.X., Sun C., Wang K., Zheng J. (2012). Heteromeric heat-sensitive transient receptor potential channels exhibit distinct temperature and chemical response. J. Biol. Chem..

[66] Baez-Nieto D., Raddatz N., Ferreira G., Gonzalez C., Latorre R., Islas L.D., Qin F. (2014). Current Topics in Membranes.

[67] Alvarez O., Gonzalez C., Latorre R. (2002). Counting channels: a tutorial guide on ion channel fluctuation analysis. Adv. Physiol. Educ..

[68] Perez-Reyes E., Cribbs L.L., Daud A., Lacerda A.E., Barclay J., Williamson M.P., Fox M., Rees M., Lee J.-H. (1998). Molecular characterization of a neuronal low-voltage-activated T-type calcium channel. Nature.

[69] Popescu G., Robert A., Howe J.R., Auerbach A. (2004). Reaction mechanism determines NMDA receptor response to repetitive stimulation. Nature.

[70] Brauchi S., Orio P., Latorre R. (2004). Clues to understanding cold sensation: thermodynamics and electrophysiological analysis of the cold receptor TRPM8. Proc. Natl. Acad. Sci. USA.

[71] Islas L.D., Sigworth F.J. (1999). Voltage sensitivity and gating charge in Shaker and Shab family potassium channels. J. Gen. Physiol..

[72] Latorre R., Brauchi S., Orta G., Zaelzer C., Vargas G. (2007). ThermoTRP channels as modular proteins with allosteric gating. Cell Calcium.

[73] GÜler A., Lee H., Lida T., Shimizu I., Tominaga M., Caterina M. (2002). Heat-evoked Activation of the Ion Channel, TRPV4. J. Neurosci.

[74] Saldaño T., Escobedo N., Marchetti J., Zea D.J., Mac Donagh J., Velez Rueda A.J., Gonik E., García Melani A., Novomisky Nechcoff J., Salas M.N. (2022). Impact of protein conformational diversity on AlphaFold predictions. Bioinformatics.

[75] Cordero-Morales J.F., Gracheva E.O., Julius D. (2011). Cytoplasmic Ankyrin Repeats of Transient Receptor Potential A1 (TRPA1) Dictate Sensitivity to Thermal and Chemical Stimuli. Proc. Natl. Acad. Sci. USA.

[76] Gaudet R. (2008). A primer on ankyrin repeat function in TRP channels and beyond. Mol. Biosyst..

[77] Ladrón-de-Guevara E., Dominguez L., Rangel-Yescas G.E., Fernández-Velasco D.A., Torres-Larios A., Rosenbaum T., Islas L.D. (2020). The Contribution of the Ankyrin Repeat Domain of TRPV1 as a Thermal Module. Biophys. J..

[78] Zheng W., Qin F. (2015). A combined coarse-grained and all-atom simulation of TRPV1 channel gating and heat activation. J. Gen. Physiol..

[79] Moparthi L., Kichko T.I., Eberhardt M., Högestätt E.D., Kjellbom P., Johanson U., Reeh P.W., Leffler A., Filipovic M.R., Zygmunt P.M. (2016). Human TRPA1 is a heat sensor displaying intrinsic U-shaped thermosensitivity. Sci. Rep..

[80] Moparthi L., Survery S., Kreir M., Simonsen C., Kjellbom P., Högestätt E.D., Johanson U., Zygmunt P.M. (2014). Human TRPA1 is intrinsically cold- and chemosensitive with and without its N-terminal ankyrin repeat domain. Proc. Natl. Acad. Sci. USA.

[81] Diver M.M., Lin King J.V., Julius D., Cheng Y. (2022). Sensory TRP Channels in Three Dimensions. Annu. Rev. Biochem..

[82] Patapoutian A., Peier A.M., Story G.M., Viswanath V. (2003). ThermoTRP channels and beyond: mechanisms of temperature sensation. Nat. Rev. Neurosci..

[83] Laursen W.J., Anderson E.O., Hoffstaetter L.J., Bagriantsev S.N., Gracheva E.O. (2015). Species-specific Temperature Sensitivity of TRPA1. Temperature (Austin).

[84] Wehner R., Marsh A.C., Wehner S. (1992). Desert ants on a thermal tightrope. Nature.

[85] Heinrich B., Heinrich B. (1981). Insect thermoregulation.

[86] Neven L.G. (2000). Physiological responses of insects to heat. Postharvest Biol. Technol..

[87] Tsai C.-C., Childers R.A., Nan Shi N., Ren C., Pelaez J.N., Bernard G.D., Pierce N.E., Yu N. (2020). Physical and behavioral adaptations to prevent overheating of the living wings of butterflies. Nat. Commun..

[88] Digby P.S.B. (1955). Factors affecting the temperature excess of insects in sunshine. J. Exp. Biol..

[89] Pincebourde S., Dillon M.E., Woods H.A. (2021). Body size determines the thermal coupling between insects and plant surfaces. Funct. Ecol..

[90] de la Vega G.J., Medone P., Ceccarelli S., Rabinovich J., Schilman P.E. (2015). Geographical distribution, climatic variability and thermo-tolerance of Chagas disease vectors. Ecography.

[91] Chestovich P.J., Saroukhanoff R.Z., Moujaes S.F., Flores C.E., Carroll J.T., Saquib S.F. (2023). Temperature Profiles of Sunlight-Exposed Surfaces in a Desert Climate: Determining the Risk for Pavement Burns. J. Burn Care Res..

[92] Abad-Franch F., Gurgel Gonçalves R., Guarneri A., Lorenzo M. (2022). Triatomina: The Biology of Chagas Disease Vectors.

[93] Heger T.J., Guerin P.M., Eugster W. (2006). Microclimatic factors influencing refugium suitability for Rhodnius prolixus. Physiol. Entomol..

[94] Marliére N.P., Latorre-Estivalis J.M., Lorenzo M.G., Carrasco D., Alves-Silva J., Rodrigues J.d.O., Ferreira L.d.L., Lara L.d.M., Lowenberger C., Guarneri A.A. (2015). Trypanosomes Modify the Behavior of Their Insect Hosts: Effects on Locomotion and on the Expression of a Related Gene. PLoS Negl. Trop. Dis..

[95] Marliére N.P., Lorenzo M.G., Guarneri A.A. (2021). Trypanosoma cruzi-infected Rhodnius prolixus endure increased predation facilitating parasite transmission to mammal hosts. PLoS Negl. Trop. Dis..

[96] Sokabe T., Tominaga M. (2009). A temperature-sensitive TRP ion channel, Painless, functions as a noxious heat sensor in fruit flies. Commun. Integr. Biol..

[97] Kim H.G., Margolies D., Park Y. (2015). The roles of thermal transient receptor potential channels in thermotactic behavior and in thermal acclimation in the red flour beetle, Tribolium castaneum. J. Insect Physiol..

[98] Zermoglio P.F., Latorre-Estivalis J.M., Crespo J.E., Lorenzo M.G., Lazzari C.R. (2015). Thermosensation and the TRPV channel in Rhodnius prolixus. J. Insect Physiol..

[99] Voets T., Talavera K., Owsianik G., Nilius B. (2005). Sensing with TRP channels. Nat. Chem. Biol..

[100] Liénard M.A., Valencia-Montoya W.A., Pierce N.E. (2022). Molecular advances to study the function, evolution and spectral tuning of arthropod visual opsins. Philos. Trans. R. Soc. Lond. B Biol. Sci..

[101] Yin C., Shen G., Guo D., Wang S., Ma X., Xiao H., Liu J., Zhang Z., Liu Y., Zhang Y. (2016). InsectBase: a resource for insect genomes and transcriptomes. Nucleic Acids Res..

[102] Thurmond J., Goodman J.L., Strelets V.B., Attrill H., Gramates L.S., Marygold S.J., Matthews B.B., Millburn G., Antonazzo G., Trovisco V. (2019). FlyBase 2.0: the next generation. Nucleic Acids Res..

[103] Elsik C.G., Tayal A., Diesh C.M., Unni D.R., Emery M.L., Nguyen H.N., Hagen D.E. (2016). Hymenoptera Genome Database: integrating genome annotations in HymenopteraMine. Nucleic Acids Res..

[104] Altschul S.F., Madden T.L., Schäffer A.A., Zhang J., Zhang Z., Miller W., Lipman D.J. (1997). Gapped BLAST and PSI-BLAST: a new generation of protein database search programs. Nucleic Acids Res..

[105] Poelchau M., Childers C., Moore G., Tsavatapalli V., Evans J., Lee C.-Y., Lin H., Lin J.-W., Hackett K. (2015). The i5k Workspace@NAL—enabling genomic data access, visualization and curation of arthropod genomes. Nucleic Acids Res..

[106] Emms D.M., Kelly S. (2019). OrthoFinder: phylogenetic orthology inference for comparative genomics. Genome Biol..

[107] Katoh K., Standley D.M. (2013). MAFFT Multiple sequence alignment software version 7: improvements in performance and usability. Mol. Biol. Evol..

[108] Nguyen L.-T., Schmidt H.A., von Haeseler A., Minh B.Q. (2015). IQ-TREE: A fast and effective stochastic algorithm for estimating maximum likelihood phylogenies. Mol. Biol. Evol..

[109] Trifinopoulos J., Nguyen L.-T., von Haeseler A., Minh B.Q. (2016). W-IQ-TREE: a fast online phylogenetic tool for maximum likelihood analysis. Nucleic Acids Res..

[110] Kalyaanamoorthy S., Minh B.Q., Wong T.K.F., von Haeseler A., Jermiin L.S. (2017). ModelFinder: Fast model selection for accurate phylogenetic estimates. Nat. Methods.

[111] Yu G. (2020). Using ggtree to Visualize Data on Tree-Like Structures. Curr. Protoc. Bioinformatics.

[112] Zhang H., Gao S., Lercher M.J., Hu S., Chen W.-H. (2012). EvolView, an online tool for visualizing, annotating and managing phylogenetic trees. Nucleic Acids Res..

[113] Martin M. (2011). Cutadapt Removes Adapter Sequences from High-Throughput Sequencing Reads. EMBnet J..

[114] Kim D., Paggi J.M., Park C., Bennett C., Salzberg S.L. (2019). Graph-based genome alignment and genotyping with HISAT2 and HISAT-genotype. Nat. Biotechnol..

[115] Pertea M., Pertea G.M., Antonescu C.M., Chang T.C., Mendell J.T., Salzberg S.L. (2015). StringTie enables improved reconstruction of a transcriptome from RNA-seq reads. Nat. Biotechnol..

[116] Bryant D.M., Johnson K., DiTommaso T., Tickle T., Couger M.B., Payzin-Dogru D., Lee T.J., Leigh N.D., Kuo T.H., Davis F.G. (2017). A Tissue-Mapped Axolotl De Novo Transcriptome Enables Identification of Limb Regeneration Factors. Cell Rep..

[117] Lee E., Helt G.A., Reese J.T., Munoz-Torres M.C., Childers C.P., Buels R.M., Stein L., Holmes I.H., Elsik C.G., Lewis S.E. (2013). Web Apollo: a web-based genomic annotation editing platform. Genome Biol..

[118] Soneson C., Matthes K.L., Nowicka M., Law C.W., Robinson M.D. (2016). Isoform prefiltering improves performance of count-based methods for analysis of differential transcript usage. Genome Biol..

[119] Kearse M., Moir R., Wilson A., Stones-Havas S., Cheung M., Sturrock S., Buxton S., Cooper A., Markowitz S., Duran C. (2012). Geneious Basic: An integrated and extendable desktop software platform for the organization and analysis of sequence data. Bioinformatics.

[120] (2015). The PyMOL Molecular Graphics System, Version 1.8, Schrödinger, LLC. https://pymol.org/support.html.

[121] Holm L. (2020). DALI and the persistence of protein shape. Protein Sci..

[122] Yao J., Liu B., Qin F. (2009). Rapid Temperature Jump by Infrared Diode Laser Irradiation for Patch-Clamp Studies. Biophys. J..

[123] Peier A.M., Moqrich A., Hergarden A.C., Reeve A.J., Andersson D.A., Story G.M., Earley T.J., Dragoni I., McIntyre P., Bevan S., Patapoutian A. (2002). A TRP channel that senses cold stimuli and menthol. Cell.

[124] Smith G.D., Gunthorpe M.J., Kelsell R.E., Hayes P.D., Reilly P., Facer P., Wright J.E., Jerman J.C., Walhin J.P., Ooi L. (2002). TRPV3 is a temperature-sensitive vanilloid receptor-like protein. Nature.

[125] Xu H., Ramsey I.S., Kotecha S.A., Moran M.M., Chong J.A., Lawson D., Ge P., Lilly J., Silos-Santiago I., Xie Y. (2002). TRPV3 is a calcium-permeable temperature-sensitive cation channel. Nature.

[126] Watanabe H., Vriens J., Suh S.H., Benham C.D., Droogmans G., Nilius B. (2002). Heat-evoked activation of TRPV4 channels in a HEK293 cell expression system and in native mouse aorta endothelial cells. J. Biol. Chem..

[127] McKemy D.D., Neuhausser W.M., Julius D. (2002). Identification of a cold receptor reveals a general role for TRP channels in thermosensation. Nature.

[128] Zimmermann K., Lennerz J.K., Hein A., Link A.S., Kaczmarek J.S., Delling M., Uysal S., Pfeifer J.D., Riccio A., Clapham D.E. (2011). Transient receptor potential cation channel, subfamily C, member 5 (TRPC5) is a cold-transducer in the peripheral nervous system. Proc. Natl. Acad. Sci. USA.

[129] Story G.M., Peier A.M., Reeve A.J., Eid S.R., Mosbacher J., Hricik T.R., Earley T.J., Hergarden A.C., Andersson D.A., Hwang S.W. (2003). ANKTM1, a TRP-like Channel Expressed in Nociceptive Neurons, Is Activated by Cold Temperatures. Cell.

[130] Saito S., Banzawa N., Fukuta N., Saito C.T., Takahashi K., Imagawa T., Ohta T., Tominaga M. (2014). Heat and noxious chemical sensor, chicken TRPA1, as a target of bird repellents and identification of its structural determinants by multispecies functional comparison. Mol. Biol. Evol..

[131] Ohkita M., Saito S., Imagawa T., Takahashi K., Tominaga M., Ohta T. (2012). Molecular Cloning and Functional Characterization of Xenopus tropicalis Frog Transient Receptor Potential Vanilloid 1 Reveal Its Functional Evolution for Heat, Acid, and Capsaicin Sensitivities in Terrestrial Vertebrates. J. Biol. Chem..

[132] Saito S., Fukuta N Fau - Shingai R., Shingai R Fau - Tominaga M., Tominaga M. (2011). Evolution of vertebrate transient receptor potential vanilloid 3 channels: Opposite Temperature Sensitivity between Mammals and Western Clawed Frogs. PLoS Genet..

[133] Gracheva E.O., Ingolia N.T., Kelly Y.M., Cordero-Morales J.F., Hollopeter G., Chesler A.T., Sánchez E.E., Perez J.C., Weissman J.S., Julius D. (2010). Molecular basis of infrared detection by snakes. Nature.

[134] Saito S., Nakatsuka K., Takahashi K., Fukuta N., Imagawa T., Ohta T., Tominaga M. (2012). Analysis of Transient Receptor Potential Ankyrin 1 (TRPA1) in Frogs and Lizards Illuminates Both Nociceptive Heat and Chemical Sensitivities and Coexpression with TRP Vanilloid 1 (TRPV1) in Ancestral Vertebrates∗. J. Biol. Chem..

[135] Akashi H.D., Saito S., Cádiz Díaz A., Makino T., Tominaga M., Kawata M. (2018). Comparisons of behavioural and TRPA1 heat sensitivities in three sympatric Cuban Anolis lizards. Mol. Ecol..

[136] Oda M., Ogino H., Kubo Y., Saitoh O. (2019). Functional properties of axolotl transient receptor potential ankyrin 1 revealed by the heterologous expression system. Neuroreport.

[137] Oda M., Kurogi M., Kubo Y., Saitoh O. (2016). Sensitivities of Two Zebrafish TRPA1 Paralogs to Chemical and Thermal Stimuli Analyzed in Heterologous Expression Systems. Chem. Senses.

[138] Oda M., Saito K., Hatta S., Kubo Y., Saitoh O. (2017). Chemical and thermal sensitivity of medaka TRPA1 analyzed in heterologous expression system. Biochem. Biophys. Res. Commun..

[139] Oda M., Kubo Y., Saitoh O. (2018). Sensitivity of Takifugu TRPA1 to thermal stimulations analyzed in oocytes expression system. Neuroreport.

[140] Viswanath V., Story G.M., Peier A.M., Petrus M.J., Lee V.M., Hwang S.W., Patapoutian A., Jegla T. (2003). Opposite thermosensor in fruitfly and mouse. Nature.

[141] Li T., Saito C.T., Hikitsuchi T., Inoguchi Y., Mitsuishi H., Saito S., Tominaga M. (2019). Diverse sensitivities of TRPA1 from different mosquito species to thermal and chemical stimuli. Sci. Rep..

