# Supplementary material for: TRPA5 encodes a thermosensitive ankyrin ion channel receptor in a triatomine insect
Source: iScience. 2024 Mar 20;27(4):109541. doi: 10.1016/j.isci.2024.109541 (PMC10993193; doi:10.1016/j.isci.2024.109541)
Supplement: Document S1. Figures S1–S10, Tables S3–S7, and Data S1 [file mmc1.pdf]

iScience, Volume *27*

Supplemental information

TRPA5 encodes a thermosensitive ankyrin ion channel receptor in a triatomine insect

Marjorie A. Liénard, David Baez-Nieto, Cheng-Chia Tsai, Wendy A. Valencia-Montoya, Balder Werin, Urban Johanson, Jean-Marc Lassance, Jen Q. Pan, Nanfang Yu, and Naomi E. Pierce

Supplemental Figures

Figure S1: Phylogeny of insect TRPA channels, related to Figure 1A.

Figure S2: Phylogeny of insect TRPA5 channels, related to Figure 1A.

Figure S3: qPCR analysis of RpTRPA5B, related to Figure 1B.

Figure S4: TRPA5B whole cell and surface expression, related to Figure 2.

Figure S5: Validation with rTRPV1 and dTRPA1-D, related to Figure 2.

Figure S6: Open pipette temperature calibration and optic fiber positioning, related to Figure 2.

Figure S7: DALI analysis, related to Figure 3.

Figure S8: Comparison of experimentally determined structure of dTRPA1 in state-2 (PDB ID 7YKS) and dTRPA1 tetrameric AlphaFold model, related to Figure 3

Figure S9: Distances between corresponding residues in the upper and lower gates of dTRPA1, hTRPA1 and RpTRPA5B, related to Figure 3

Figure S10: Comparison of 6V9Y and RpTRPA5, related to Figure 3

Supplemental Tables

Table S3: qPCR data for RpTRPA5B tissue expression analysis, related to Figure S3

Table S4: Cell density for whole patch recordings, related to Figure 2 and Figure S5

Table S5: Ankyrin repeats in monomeric structure models of TRPAs, related to Figure 3

Table S6: Comparison of residues in the upper and lower gate between the AlphaFold tetrameric model and determined structures of dTRPA1, related to Figure S9

Table S7: Oligonucleotide primer sequences, related to Figure 2, S3, S5

Supplemental file

Data S1: List of all source data, related to Figure 1,2,3, and Figures S1 to S10.

Figure S1: Phylogeny of insect TRPA channels, related to Figure 1A.

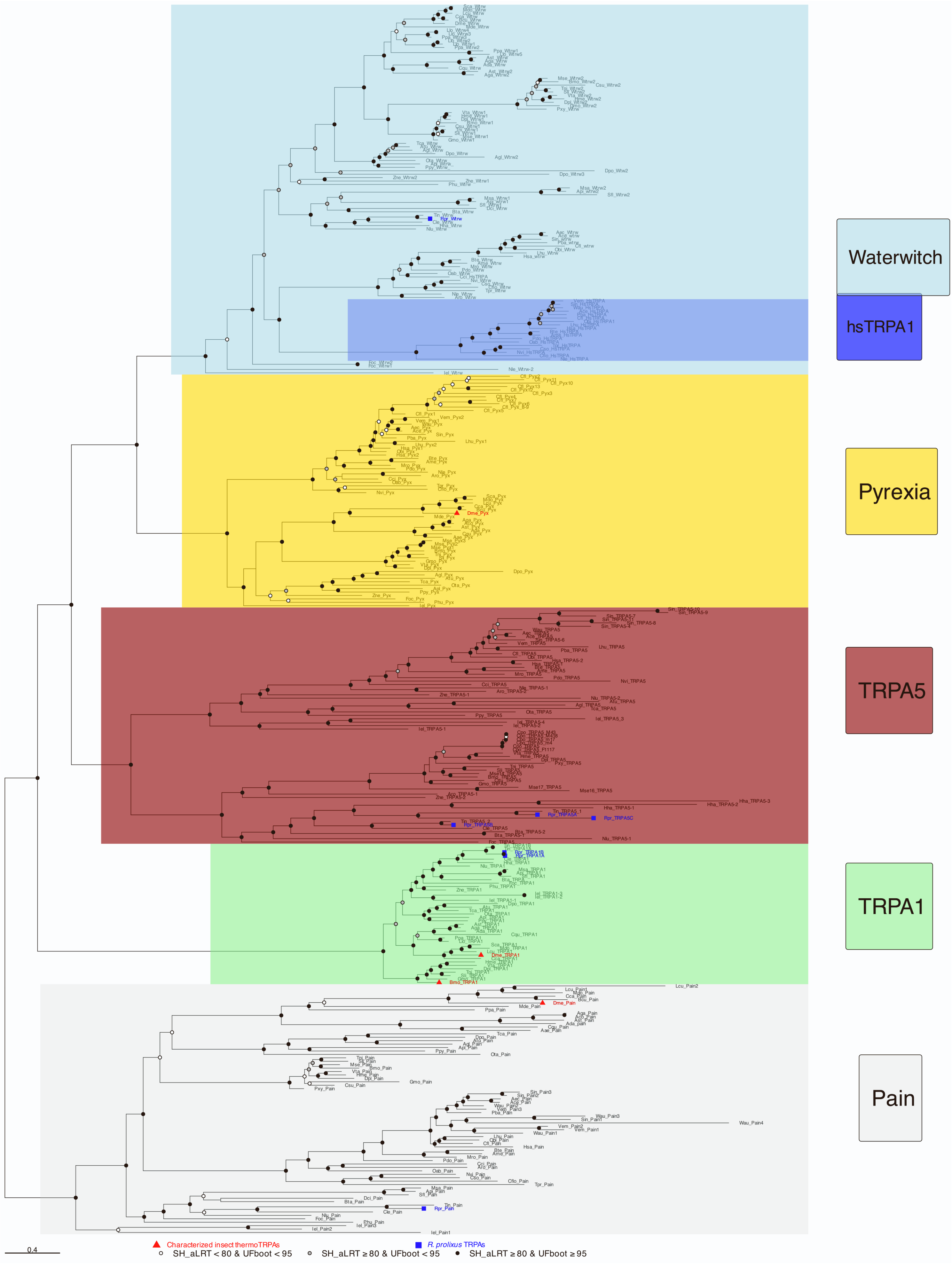

Figure S1. Phylogeny of insect TRPA channels, related to Figure 1A. The Maximum-likelihood phylogeny of amino acid sequences includes representative insect ion channel members of the TRPA1, Pain, Pyrexia, hymenoptera-specific TRPA (hsTRPA), TRPA5 and Waterwitch (Wtw) subfamilies. Accession numbers are listed in Table S1. The tree was inferred in IQ-TREE v1.6.11 using ModelFinder, Ultrafast Bootstrap (UFboot), 1000 replicates, using a best-fit model JTT+F+I+G4 measured by the Bayesian information criterion (BIC). Branches were assigned Shimodaira-Hasegawa approximate likelihood ratio test (SH-aLRT) and UFboot supports. The tree was visualized in Rstudio (2021.09.2) using ggtree (Source data Figure S1). Species represented are **Anoplura**: *Pediculus humanus* (Phu, head louse), **Coleoptera**: *Tribolium castaneum* (Tca, red flour beetle), *Dendroctonus ponderosae* (Dpo, Mountain pine beetle), *Photynus pyralis* (Ppy, Common Eastern Firefly), *Agrilus planipennis* (Apl, Emerald ash borer), *Anoplophora glabripennis* (All, Asian longhorned beetle), *Aethina tumida* (Aut, Small hive beetle), *Onthophagus taurus* (Ota, bull dung beetle), **Diptera**: *Drosophila melanogaster* (Dme, fruitfly), *Mayetiola destructor* (Mde, Hessian fly), *Culex quinquefasciatus* (Cqu, Southern house mosquito), *Anopheles stephensi* (Ast), *Anopheles gambiae* (Aga), *Anopheles darlingi* (Ada), *Bactrocera cucurbitae* (Bcu, Melon fly), *Ceratitis capitata* (Cca, Mediterranean fruit fly), *Musca domestica* (Mdo, housefly), *Lutzomyia longipalpis (Llo, Sandfly), Phlebotomus papatasi* (Ppa, Sandfly), *Stomoxys calcitrans* (Sca, Barn fly), **Hemiptera:** *Acyrthosiphon pisum* (Api, pea aphid)*, Nilaparvata lugens* (Nlu, Brown planthopper), *Diaphorina citri* (Dci, Asiatic citrus psyllid), *Cimex lectularius* (Cle, Bed bug)*, Rhodnius prolixus* (Rpr, Kissing bug), *Triatoma infestans* (Tin, Winchuka); **Hymenoptera:** *Apis mellifera* (Ame, Western honeybee) *, Bombus terrestris* (Bte, Buff-tailed bumblebee)*, Megachile rotundata* (Mro, Alfalfa leaf cutting bee), *Nasonia vitripenis* (Nvi, Jewel wasp), *Harpegnathos saltator* (Hsa, Jumping ant)*, Linepithema humile* (Lhu, Argentine ant), *Campanatus floridanus* (Cfl, Florida carpenter ant), *Pogonomyrmex barbatus* (Pba, red harvester ant), *Atta cephalotes* (Ace, Leafcutter ant), *Acromyrmex echinatior* (Aec, Panamian leafctutter ant), *Solenopsis invicta* (Sin, Red imported fire ant)*, Vollenhovia emeryi* (Vem, ant), *Athalia rosae* (Aro, Turnip sawfly), *Cerapachys biroi* (Cbi, Clonal raider ant), *Ceratosolen solmsi* (Cso, Fig wasp), *Wasmannia auropunctata* (Wau, Electric ant), **Isoptera:** *Zootermopsis nevadensis* (Zne, Dampwood termite), **Lepidoptera**: *Bombyx mori* (Bmo, Silkmoth)*, Chilo suppressalis* (Csu, Asiatic rice borer), *Danaus plexippus* (Dpl, Monarch butterfly), *Heliconius melpomene* (Hme, Postman butterfly), *Plutella xylostella* (Diamondback moth), *Manduca sexta* (Mse, Tobacco hornworm) *Cydia pomonella* (Cpo, Codling moth), **Odonata**: *Ischnura elegans* (Iel, Bluetail damselfly). See also Table S1.

Figure S2: Phylogeny of insect TRPA5 channels, related to Figure 1A.

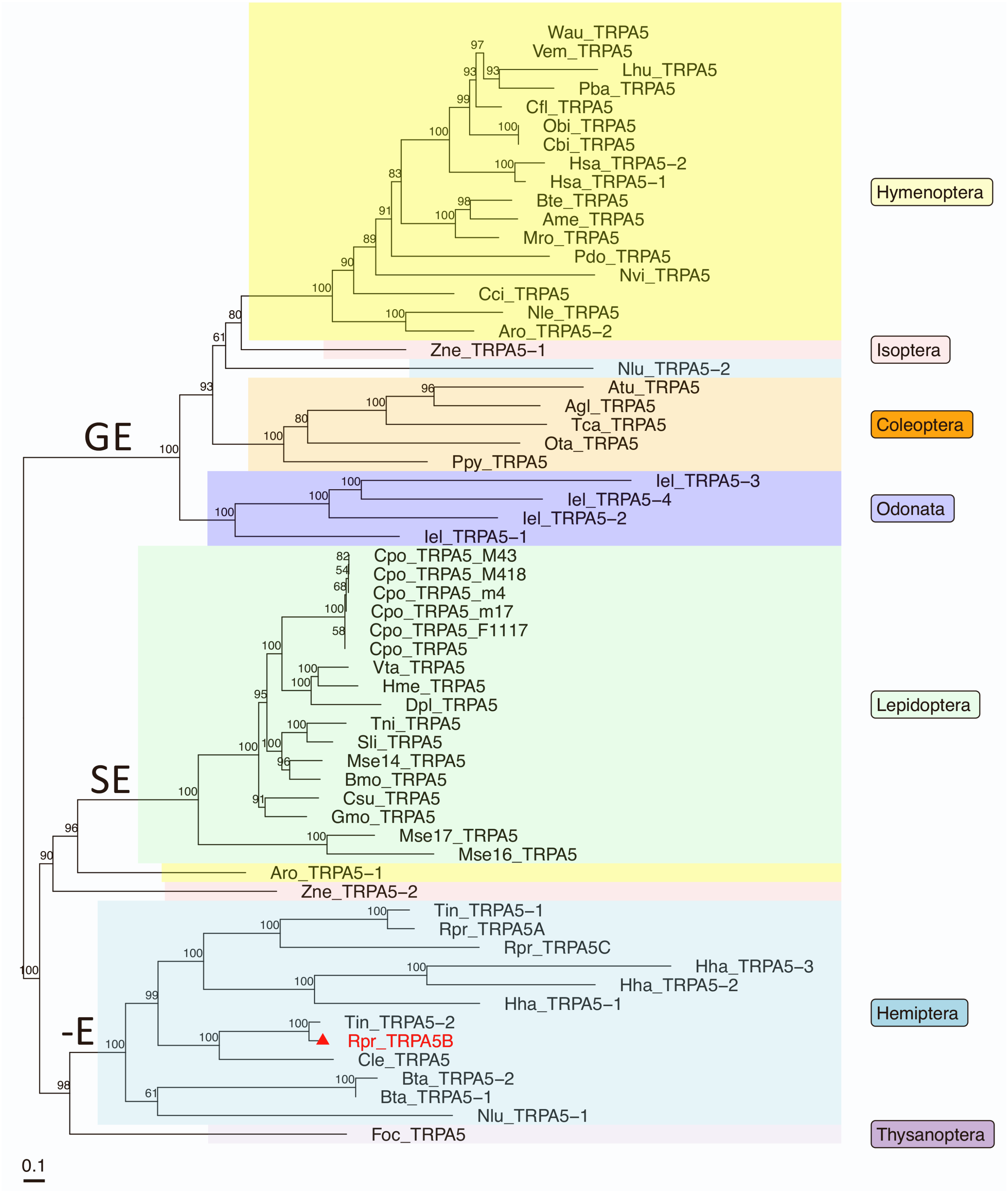

Figure S2. Phylogeny of insect TRPA5 channels, related to Figure 1A. TRPA5 channels are present across insect Orders but Diptera. The Maximum-likelihood phylogeny of amino acid sequences includes representative ion channel members of the TRPA5 subfamily. The tree was inferred in IQ-TREE v1.6.11 using ModelFinder, Ultrafast Bootstrap (UFboot), 1000 replicates, using a best-fit model JTT+F+I+G4 measured by the Bayesian information criterion (BIC). Branches were assigned Shimodaira-Hasegawa approximate likelihood ratio test (SH-aLRT) and UFboot supports. The tree was visualized in Rstudio (2021.09.2) using ggtree (see also Source data for Figure S2 in the key reousrce table). Species abbreviations are as follows: **Coleoptera:** *Tribolium castaneum* (Tca, red flour beetle), *Photynus pyralis* (Ppy, Common Eastern Firefly), *Anoplophora glabripennis* (Agl, Asian longhorned beetle), *Aethina tumida* (Aut, Small hive beetle), *Onthophagus taurus* (Ota, bull dung beetle); **Hemiptera**: *Bemisia tabaci* (Bta, Silverleaf Whitefly), *Cimex lectularius* (Cle, Bed bug), *Halyomorpha halys* (Hha, Brown marmorated stinkbug), *Nilaparvata lugens* (Nlu, Brown planthopper), *Rhodnius prolixus* (Rpr, Kissing bug), *Triatoma infestans* (Tin, Winchuka); **Hymenoptera**: *Apis mellifera* (Ame, Western honeybee) , *Athalia rosae* (Aro, Turnip sawfly), *Bombus terrestris* (Bte, Buff-tailed bumblebee), *Megachile rotundata* (Mro, Alfalfa leaf cutting bee), *Nasonia vitripennis* (Nvi, Jewel wasp), *Harpegnathos saltator* (Hsa, Jumping ant), *Linepithema humile* (Lhu, Argentine ant), *Campanatus floridanus* (Cfl, Florida carpenter ant), *Pogonomyrmex barbatus* (Pba, red harvester ant), *Atta cephalotes* (Ace, Leafcutter ant), *Acromyrmex echinatior* (Aec, Panamian leafcutter ant), *Solenopsis invicta* (Sin, Red imported fire ant), *Vollenhovia emery* (Vem, ant), *Ooceraea biroi* (Cbi, Clonal raider ant), *Wasmannia auropunctata* (Wau, Electric ant), *Polisted dominula* (Pdo, European paper wasp), *Neodiprion lecontei* (Nle, Red-headed pine sawfly), *Cephus cinctus* (Cci, Wheat stem sawfly); **Isoptera**: *Zootermopsis nevadensis* (Zne, Dampwood termite); **Lepidoptera:** *Bombyx mori* (Bmo, Silkmoth), *Chilo suppressalis* (Csu, Asiatic rice borer), *Danaus plexippus* (Dpl, Monarch butterfly), *Heliconius melpomene* (Hme, Postman butterfly), *Plutella xylostella* (Pxy, Diamondback moth), *Manduca sexta* (Mse, Tobacco hornworm), *Cydia pomonella* (Cpo, Codling moth), *Vanessa tameamea* (Vta, Kamehameha butterfly), *Trichoplusia ni* (Tni, Cabbage looper), *Spodoptera litura* (Sli, Tobacco cutworm), *Galleria mellonella* (Gmo, Greater wax moth), **Odonata**: *Ischnura elegans* (Iel, damselfly), **Thysanoptera**: *Frankliniella occidentalis* (Foc, thrips). Capital letters above branches represent the amino acid residues at the ion channel selectivity filter. See also main text and Figure 3D.

Figure S3. qPCR analysis of RpTRPA5B, related to Figure 1B.

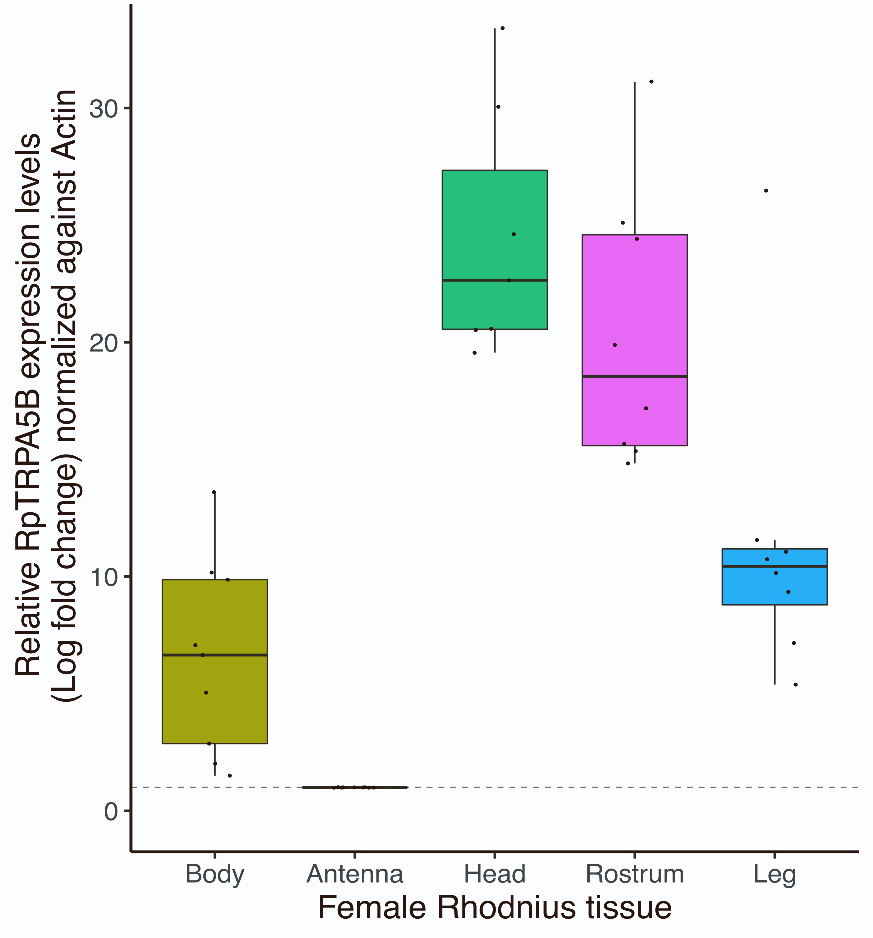

Figure S3. qPCR analysis of RpTRPA5B, related to Figure 1B. Log_2 fold change expression was monitored by quantitative PCR and calculated from raw cycle threshold (Ct) values (±SEM, n= 9). Body tissue corresponds to Abdomen and Thorax minus legs. Box-and-whisker plots were visualized in RStudio 2021.09.2. Center horizontal bars show medians, box limit indicates upper and lower quartiles, whiskers show sample 1.5x interquartile range. Expression is calibrated using Antenna (log fold change =1) and normalized against Actin. See also Table S3 and source data for Figure S3 in the key resource table.

Figure S4: TRP whole cell and surface expression, related to Figure 2.

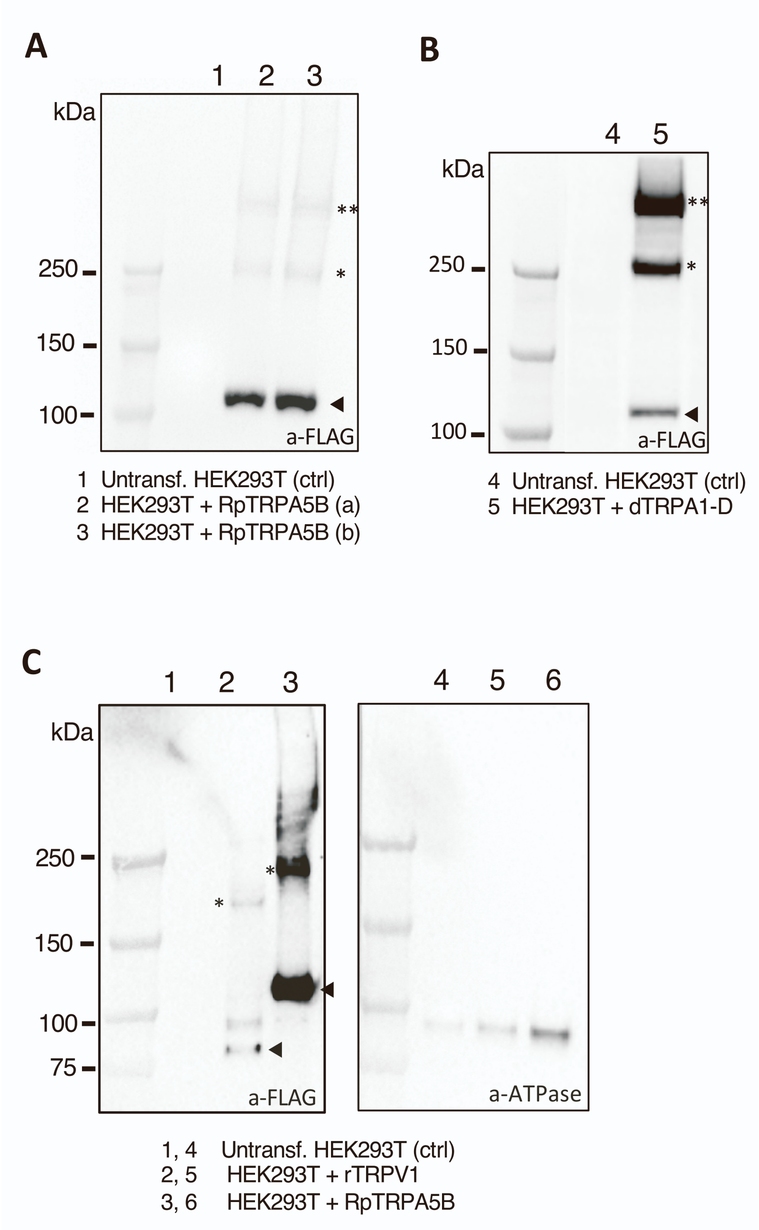

Figure S4. TRP whole cell and surface expression, related to Figure 2. A-B. RpTRPA5B anti-FLAG signals in whole cell lysates. Untransfected HEK293T cells (lanes 1,4) and HEK293T cells transfected with 2.5 µg pcDNA-FLAG-T2A-mRuby plasmid DNAs for (**A**) RpTRPA5B (a and b are two separate lysate samples, lanes 2,3), and (**B**) fruit fly dTRPA1-D (lane 5). Cells were collected 72 h after transfection. The protein ladder image taken from the same membrane is juxtaposed to the left of the immunoblot. One and two asterisks represent predicted dimeric and tetrameric TRP forms, respectively. The predicted monomeric molecular weight is indicated with a black arrowhead: RpTRPA5B, 127.78 kDa; fruit fly dTRPA1-D 138.82 kDa. **C.** Surface expression analysis of RpTRPA5B. Biotinylated surface protein eluates were run in parallel wells on the same SDS-PAGE gel, and after protein transfer, the membrane was split to probe TRP with a-FLAG (left) and ATPase with a-ATPase (right). Anti-FLAG levels in surface protein fraction are from non-transfected HEK293T cells (lane 1), cells expressing rTRPV1 (lane 2), and cells expressing RpTRPA5B (lane 3). Lanes 4 to 6 are the corresponding anti-ATPase biotin-surface fraction from non-transfected HEK293T cells (lane 4), cells expressing rTRPV1 (lane 5), and cells expressing RpTRPA5B (lane 6). One and two asterisks represent predicted dimeric and tetrameric TRP forms, respectively. The predicted monomeric weight is indicated with a black arrowhead (rTRPV1, 94.95 kDa; RpTRPA5B, 127.78 kDa). See also Figure 2, and source data for Figure S4 in the key resource table.

Figure S5: Validation with rTRPV1 and dTRPA1-D, related to Figure 2.

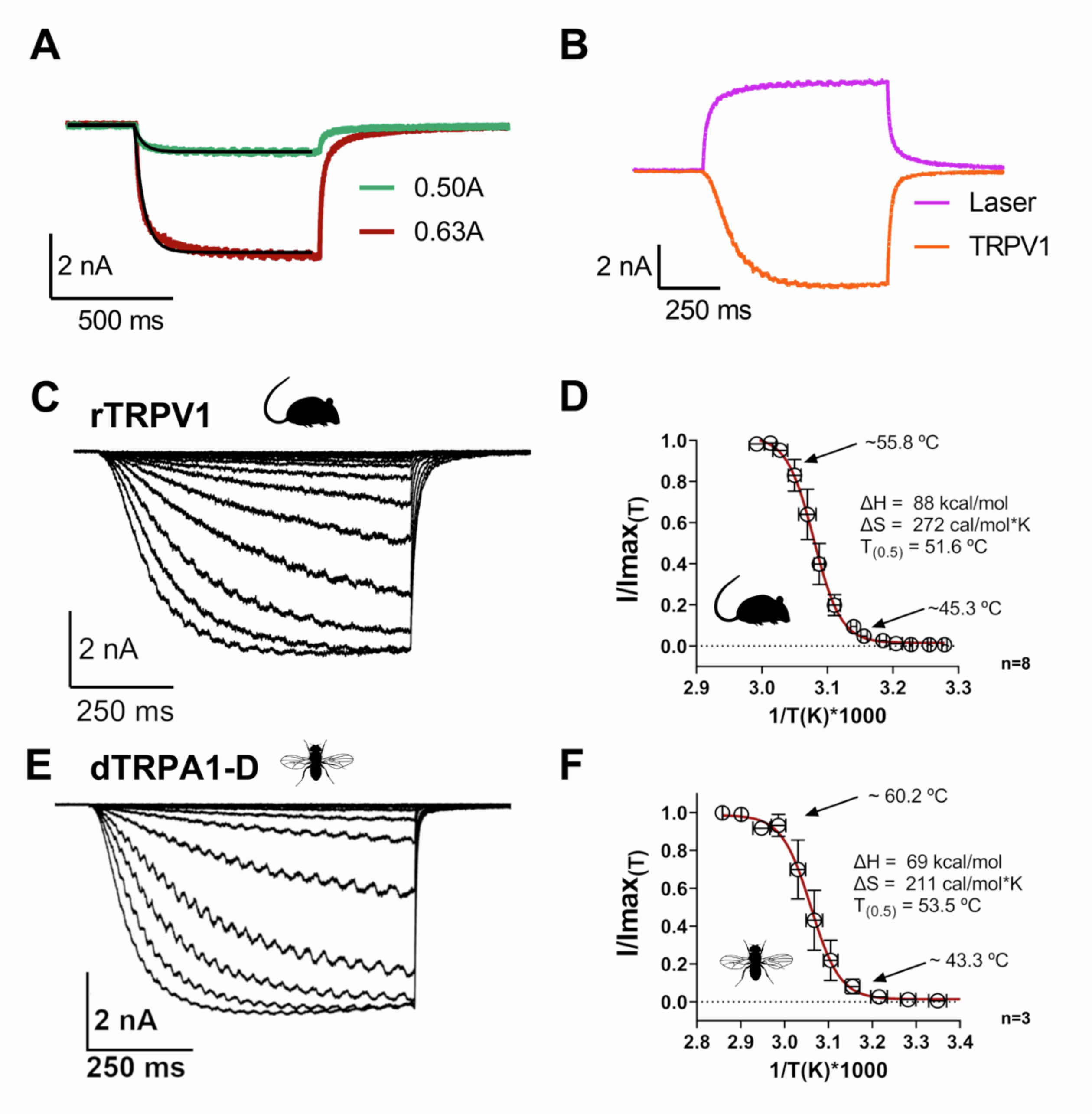

Figure S5. Validation with rTRPV1 and dTRPA1-D, related to Figure 2. A. Time course of the ionic current through the open pipette at -10 mV holding voltage when a current of 0.50A (green trace) and 0.63A (red trace) was applied to the laser diode. The temperature jumps for those currents correspond to 32.1 °C and 59.7 °C, from 22.6 °C, respectively. Both temperature jumps can be fitted by a mono-exponential function with a time constant of ~35 ms (black line). **B.** Time course comparison between the current through the open pipette (magenta trace) and a HEK293T cell expressing rTRPV1 channels under voltage clamp at -30 mV (orange trace), in response to a temperature jump from 22.6 °C to 59.7 °C (0.63A). The current through rTRPV1 channels is three times slower than the laser kinetics with a time constant of ~100 ms. The sinusoidal pattern observed within the current curves is inherent to the cyclic modulation of the laser's rapid 'on-off' cycles. **C.** Representative heat-activated current traces of a HEK293T cell expressing rTRPV1 under voltage clamp (holding voltage of -30 mV). The currents were elicited by temperature jumps from room temperature (22.6 °C) up to 59.7 °C with a duration of 700 ms. **D.** Fraction of rTRPV1 channels in the open state (Open probability, Po) as a function of the

temperature. The Po vs 1/T was fitted to a Boltzman function (red line) with the midpoint of activation ($T_{0.5}$) reached at 51.6 °C. The van't Hoff plot estimates for rTRPV1 provides an activation enthalpy of the endothermic transition at 88.3 ± 9.4 kcal/mol and an entropic change associated to the temperature activation process at 271 ± 28 cal/mol*K. at -30 mV as a function of the temperature. These values in our expression cassette are very close to previously published values of ΔH = 85 kcal/mol, $T_{0.5}$ = 51 °C [S1, S2]. These temperature values align with those reported in earlier studies for rTRPV1. The published threshold of activation for rTRPV1 is in the range 40-42 °C and corresponds to the temperature at which first observable currents are detected [S1, S3, S4]. The channel emerging temperature recorded in our setup is at 40.9 °C when the first activation currents emerge over the dotted line (**D**). We calculated values of $T_{0.1}$ (i.e., the temperature for which there is a probability for 10% of channels to be open) at 45.3 °C (-30 mV), and $T_{0.5}$ at 51.6 °C (-30 mV), consistently with values reported in Yao et al 2010 ($T_{0.5}$ = 51°C, -60 mV) [S2]. **E.** Representative heat-activated current traces of a HEK293T cell expressing dTRPA1-D channels under voltage clamp (holding voltage of -30 mV). The currents were elicited by temperature jumps from room temperature (19.4 °C) up to 63.5 °C with a duration of 700 ms. **F.** Fraction of dTRPA1-D channels in the open state (Open probability, Po) as a function of the temperature. The Po vs 1/T was fitted to a Boltzman function (red line) with the midpoint of activation ($T_{0.5}$) reached at 53.5 °C, the van't Hoff plot estimates for dTRPA1-D an activation enthalpy of the endothermic transition at 68.7 ± 13.1 kcal/mol and an entropic change associated to the temperature activation process at 211± 40 cal/mol·K at -30 mV. The stationary current at the end of the temperature pulse (last 100 ms) was used to calculate all the thermodynamics parameters. Data are presented as means ± standard deviation.

Figure S6: Open pipette calibration and fiber positioning, related to Figure 2.

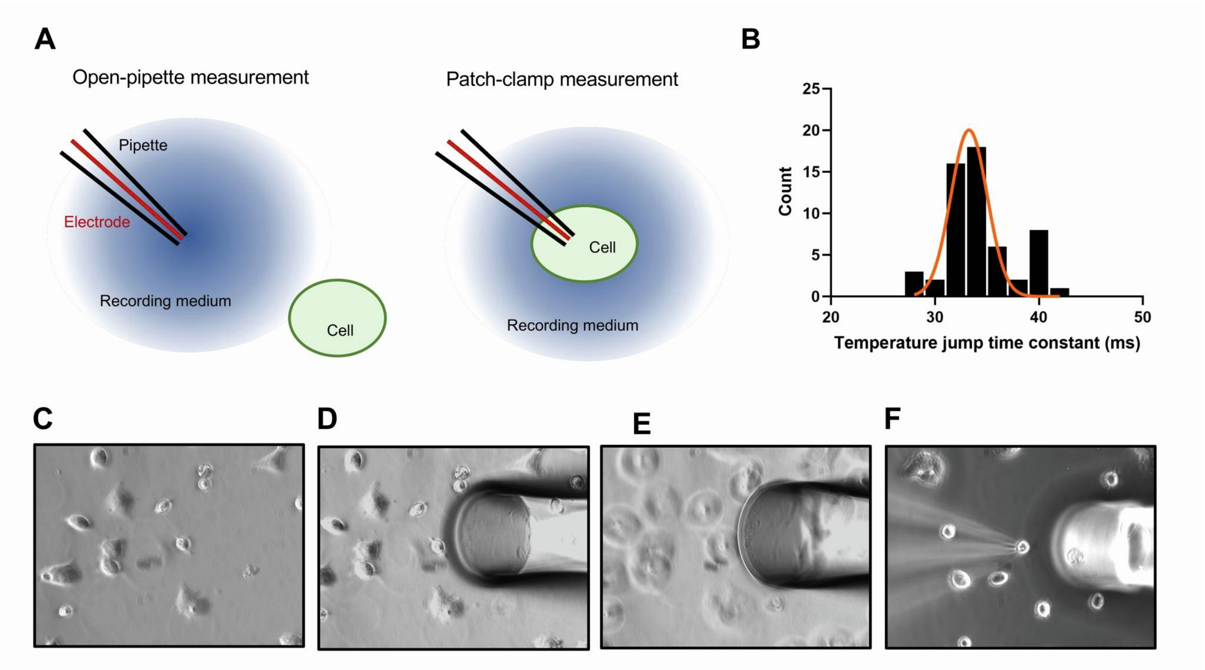

Figure S6. Open pipette temperature calibration and optic fiber positioning, related to Figure 2. **A.** Schematic representation of open pipette temperature calibration performed prior to each cell patch clamp experiment. First the open pipette is positioned under the center of the laser using the reference marks on the screen. Then we proceed to record the current through the open pipette elicited as a function of a series of near IR laser pulses. With these current traces we can estimate the temperature jumps magnitude that will be applied later to the target cell. After this the target cell is positioned in the center of the laser beam using the microscope stage translation (the optic fiber stays fixed). Once the cell is in the right position, we use the same pipette used for the calibration to carry out the electrophysiological recording, applying the same set of pulses used in the calibration **B.** Histogram of the time constants from the exponential fit to the open pipette current traces from the temperature jumps used in the rTRPV1 experiments (n=56), the mean time constant was 33.3 ± 1.8 ms. **C.** View of HEK293T cells seeded at low density on a glass cover in the recording chamber on the patch-clamp rig station. **D.** After finding a target cell co-expressing mRuby2 as a fluorescent marker, with the help of an automatized micromanipulator, the optic fiber is placed in the recording solution using the reference marks on the computer screen (not shown here) defined during the calibration with the visible laser. The depth in the solution is adjusted so that the fiber is directly above a single cell in the recording medium. **E.** The field of view is changed to align the patch clamp electrode to the reference marks. **F.** The target cell expressing mRuby2 is patched with the recording electrode, now visible on the left side, whereas the fiber is directly above the target cell. The relative position between the recording pipette and the fiber was established using a visible laser during the setup of the system, and is constant for all the experiments. See also STAR Methods.

Figure S7: DALI Analysis, related to Figure 3.

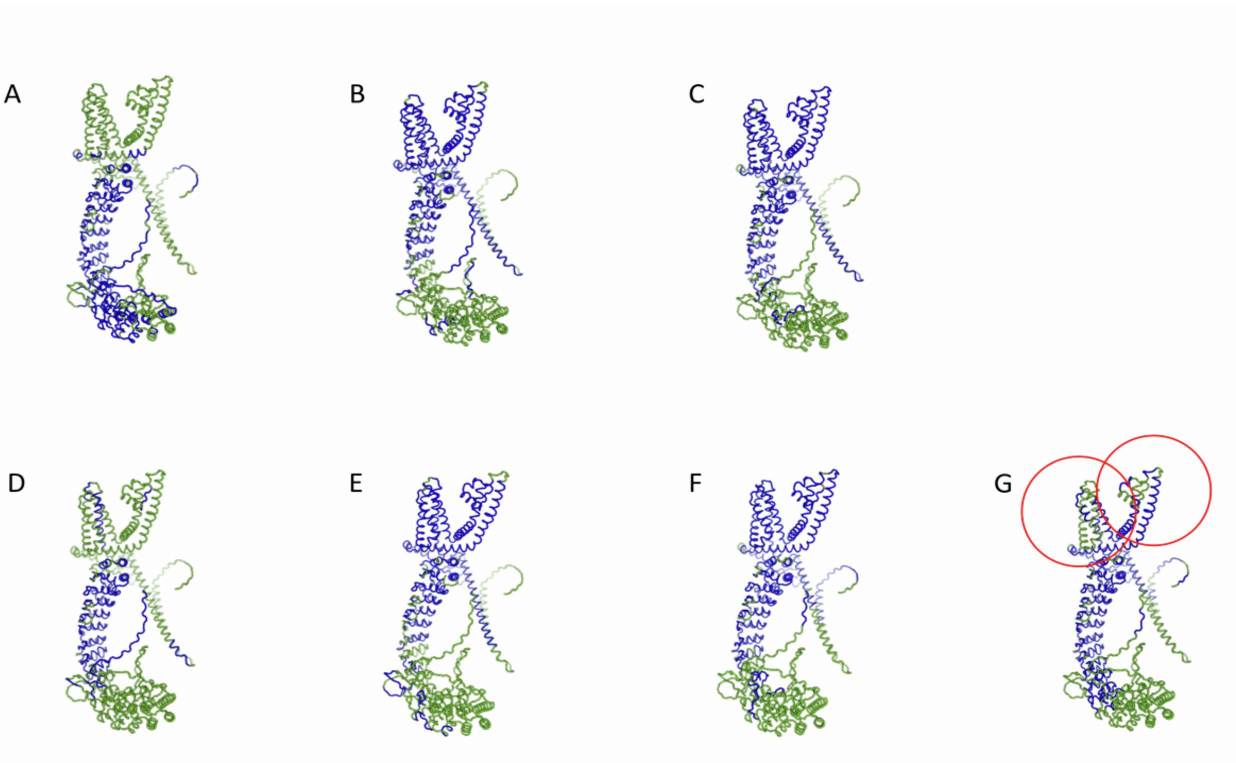

Figure S7. DALI Analysis, related to Figure 3. Pairwise structural alignment using the Dali server (http://ekhidna2.biocenter.helsinki.fi/dali/), of different monomers against *Rhodnius* TRPA5B. **A.** *Rhodnius* TRPA1, **B**. *Rhodnius* Painless, **C.** *Rhodnius* Waterwitch, **D.** *Drosophila* TRPA1, **E.** *Drosophila* Painless, **F.** *Drosophila* Waterwitch, **G.** *Drosophila* Pyrexia. The blue and green colors indicate regions with high and low structural similarity based on Z-values. The red circles in **G** indicate areas where the Pyrexia monomer stands out from Painless and Waterwitch, in that the pore region and the voltage-sensing like domain are less similar to TRPA5B. However, compared to Painless and Waterwitch the similarity in the ankyrin repeat domain (ARD) reaches further towards the N-terminus.

Figure S8: Validation of a tetrameric AlphaFold model of dTRPA1 with the experimentally determined structure dTRPA1 in state-2 (PDB ID 7YKS), related to Figure 3.

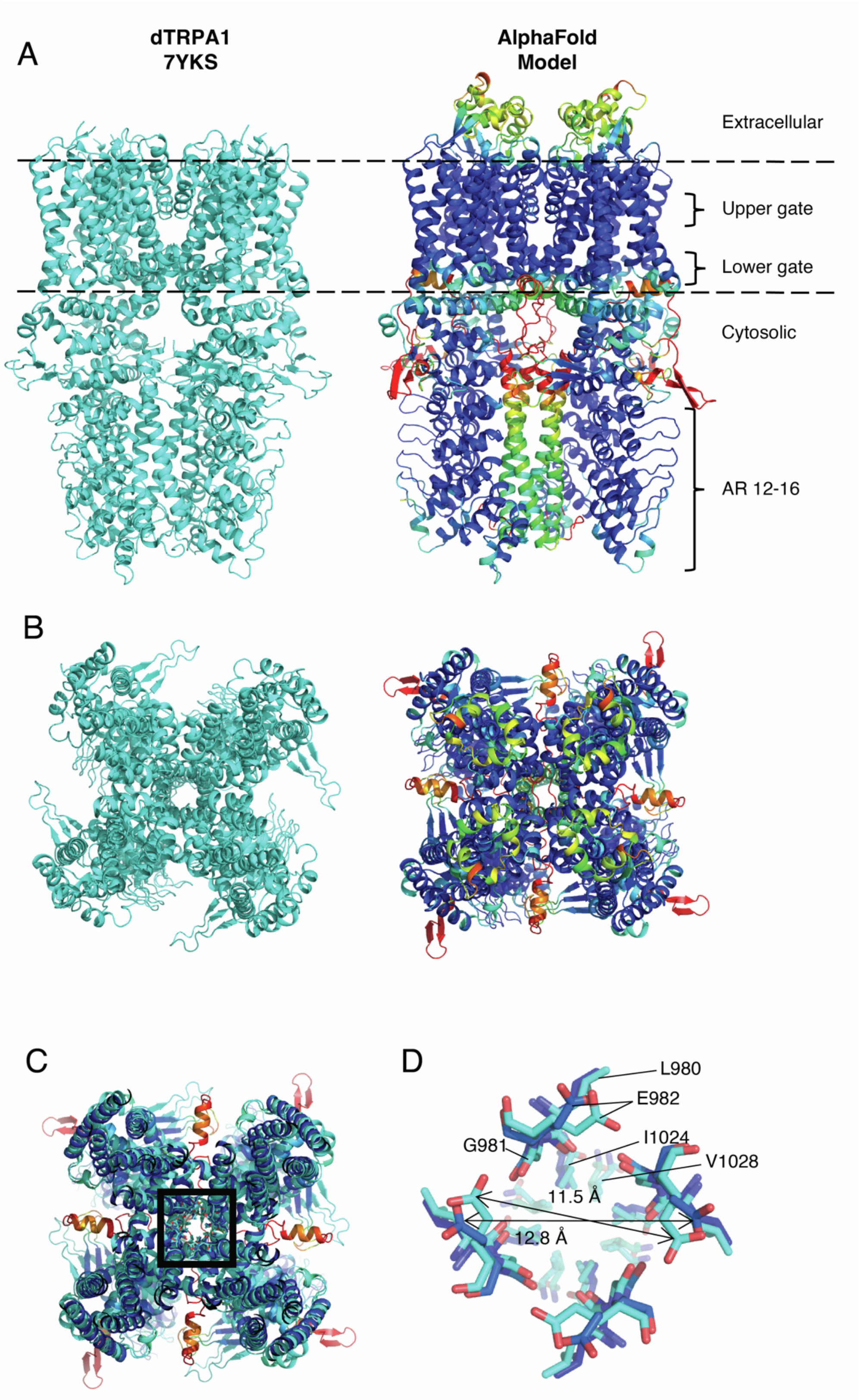

Figure S8. Validation of a tetrameric AlphaFold model of dTRPA1 with the experimentally determined structure dTRPA1 in state-2 (PDB ID 7YKS), related to Figure 3. **A**. Side view of dTRPA1 with approximate boundaries of the transmembrane domain indicated by dashed lines. The N- and C-terminal regions that are not resolved in 7YKS were excluded in the prediction. Only the last five of the 17 ankyrin repeats (AR12-16) are visible in the structure and overall regions with low confidence in the model (red-yellow) are not resolved in the structure. **B.** Top view of **A. C.** Cross section of the pore region of the aligned structure and model in the same orientation as in B. Residues in the upper and lower gate regions are shown as sticks in the central box (numbering in 7YKS L980, G981, E982, and I1024, V1028, respectively). **D**. Close up of residues shown as sticks in **C**. The spatial location of these residues is very similar in the structure and the model. The main difference being the side chain of E982, which has a slightly more distal orientation relative to the center of the pore in the model. The distance between opposite Cδ of E982 is 12.8 Å, which is similar to the distances in dTRPA1 in state 2 (7YKS, 11.5 Å) and state-1 (7YKR, 12.7 Å). RMSD of residues in the upper and lower gate of the model with determined structures of dTRPA1 are provided in Table S6.

Figure S9: Distances between corresponding residues in the upper and lower gate, related to Figure 3.

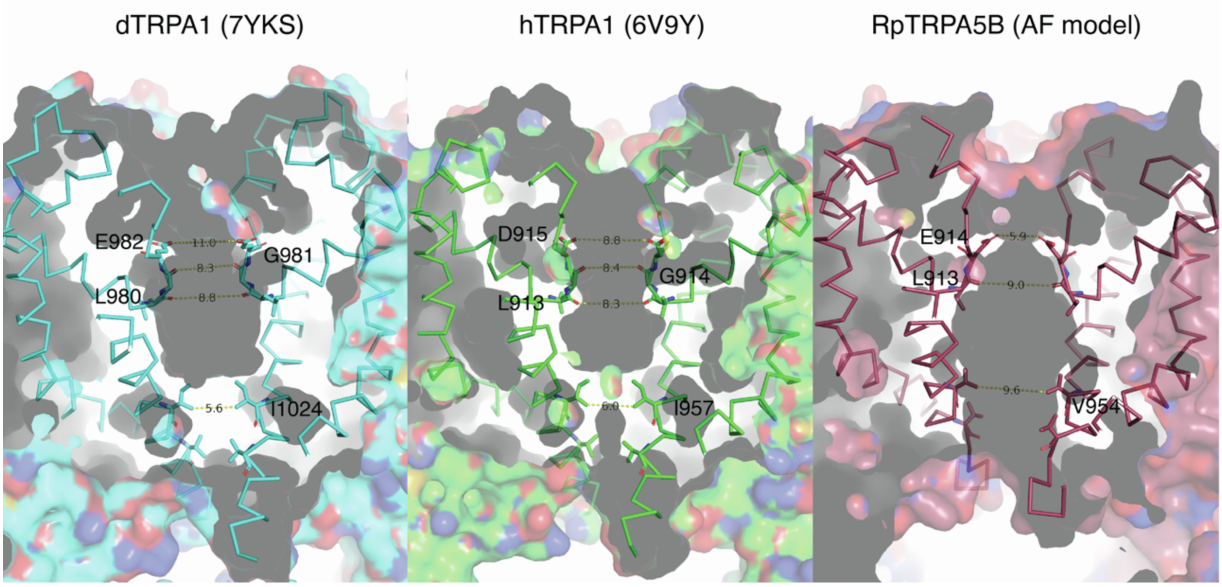

Figure S9. **Distances between corresponding residues in the upper and lower gate, related to Figure 3**. Distances between corresponding residues in the upper and lower gate in structures of TRPA1 from *Drosophila melanogaster* (dTRPA1; left) and *Homo sapiens* (hTRPA1; center) and the model of *Rhodnius prolixus*, RpTRPA5B (right).

Figure S10: Comparison of wild-type and predicted mutant RpTRPA5B, related to Figure 3.

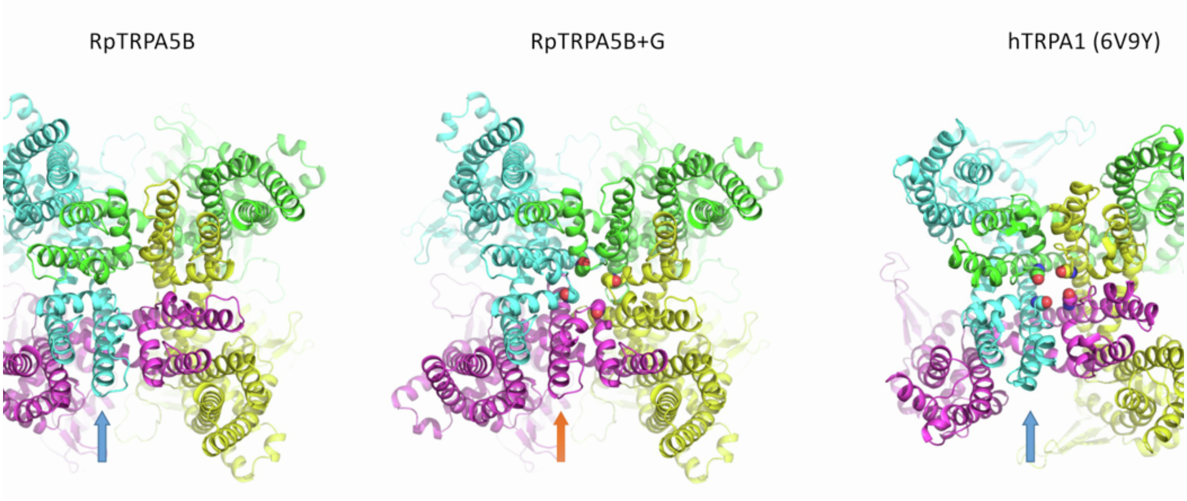

Figure S10. Comparison of wild-type and predicted mutant RpTRPA5B, related to Figure 3. Extracellular view of truncated tetrameric AlphaFold model of RpTRPA5B (left), model of the corresponding mutated RpTRPA5B with the inserted glycine (spheres; middle) and hTRPA1 (6V9Y; corresponding glycine as spheres; right). With the extra glycine the overall fold of TRPA5B is predicted to be compromised and deviates from the generic fold of TRPs that is exemplified by hTRPA. In the mutant protein, helix S6 (orange arrow) is adopting the position of S6 (blue arrow) in a neighbouring monomer in the wild type. Thus, the loop between pore helices 1 and 2 forming the selectivity filter is predicted to be abnormally placed if adding the glycine without considering the potential impact of co-evolving sites. Cartoon representation coloured according to chain.

Table S3: qPCR data for RpTRPA5B tissue expression analysis, related to Figure S3

	Exp 1		Exp 2		Exp 3	
**Tissue Type (adult female)**	**Ct Actin**	**Ct target gene TRPA5B**	**Ct Actin**	**Ct target gene TRPA5B**	**Ct Actin**	**Ct target gene TRPA5B**
	23,33	28,41	24,01	28,47	26,22	29,15
Antenna	23,39	28,37	24,43	27,75	25,49	27,89
	23,18	27,98	25,23	26,77	24,88	26,96
	17,05	25,3	19,98	25,56	18,36	26,43
Leg	16,94	25,38	18,89	25,25	18,18	25,38
	16,91	25,07	18,78	24,96	18,34	25,14
	18,85	28,06	21,2	28,09	20,35	28,22
Head	18,59	28,09	19,39	27,25	20,19	27,73
	18,54	28,25	19,72	26,83	20,17	27,56
	18,24	27,99	19,55	27,58	20,86	27,99
Rostrum	18,31	27,31	19,39	26,65	20,12	26,68
	18,26	27,6	19,12	27,11	20,31	26,82
	18,49	26,42	21,43	26,3	20,37	26,32
Thorax + Abdomen	18,47	26,29	21,21	25,15	20,4	26,07
	18,5	26,47	21,48	25,11	20,32	25,56

Tissue Type	Mean Ct Actin Exp 1	Delta Ct target Exp1			ddCT Exp 1			Log fold change E^(-ddCt) Exp 1		
		**Rep 1**	**Rep 2**	**Rep 3**	**Rep 1**	**Rep 2**	**Rep 3**	**Rep 1**	**Rep 2**	**Rep 3**
Antenna	23,30	5,11	5,07	4,68	0,00	0,00	0,00	1,00	1,00	1,00
Leg	16,97	8,33	8,41	8,10	-3,22	-3,34	-3,42	9,34	10,15	10,73
Head	18,66	9,40	9,43	9,59	-4,29	-4,36	-4,91	19,56	20,53	30,06
Rostrum	18,27	9,72	9,04	9,33	-4,61	-3,97	-4,65	24,42	15,67	25,11
Thorax + Abdomen	18,49	7,93	7,80	7,98	-2,82	-2,73	-3,30	7,08	6,65	9,87

Tissue Type	Mean Ct Actin Exp 2	Delta Ct target Exp 2			ddCT Exp 2			Log fold change E^(-ddCt) Exp 2		
		**Rep 1**	**Rep 2**	**Rep 3**	**Rep 1**	**Rep 2**	**Rep 3**	**Rep 1**	**Rep 2**	**Rep 3**
Antenna	24,56	3,91	3,19	2,21	0,00	0,00	0,00	1,00	1,00	1,00
Leg	19,22	6,34	6,03	5,74	-2,43	-2,84	-3,53	5,39	7,16	11,55
Head	19,56	8,54	7,70	7,28	-4,62	-4,50	-5,06	24,62	22,65	33,40
Rostrum	19,35	8,23	7,30	7,76	-4,31	-4,10	-5,54	19,88	17,19	46,63
Thorax + Abdomen	21,37	4,93	3,78	3,74	-1,01	-0,58	-1,52	2,02	1,50	2,87

Tissue Type	Mean Ct Actin Exp 3	Delta Ct target Exp 3			ddCT Exp 3			Log fold change E^(-ddCt) Exp 3		
		**Rep 1**	**Rep 2**	**Rep 3**	**Rep 1**	**Rep 2**	**Rep 3**	**Rep 1**	**Rep 2**	**Rep 3**
Antenna	25,53	3,62	2,36	1,43	0,00	0,00	0,00	1,00	1,00	1,00
Leg	18,29	7,09	7,09	6,85	-3,47	-4,73	-5,42	11,06	26,48	42,71
Head	20,24	7,98	7,49	7,32	-4,36	-5,13	-5,89	20,58	35,10	59,44
Rostrum	20,43	7,56	6,25	6,39	-3,94	-3,89	-4,96	15,35	14,83	31,12
Thorax + Abdomen	20,36	5,96	5,71	5,20	-2,34	-3,35	-3,77	5,05	10,17	13,61

Exp #	Antenna	Leg	Head	Rostrum	Abdomen
1.1	1,00	9,34	19,56	24,42	7,08
1.2	1,00	10,15	20,53	15,67	6,65
1.3	1,00	10,73	30,06	25,11	9,87
2.1	1,00	5,39	24,62	19,88	2,02
2.2	1,00	7,16	22,65	17,19	1,50
2.3	1,00	11,55	33,40	46,63	2,87
3.1	1,00	11,06	20,58	15,35	5,05
3.2	1,00	26,48	35,10	14,83	10,17
3.3	1,00	42,71	59,44	31,12	13,61

	Antenna	Leg	Head	Rostrum	Abdomen
**Mean tissue expresssion**	1,00	14,95	29,55	23,36	6,54
**SEM**	0	4,91	5,15	4,21	1,68

Table S4: Cell density for whole patch recordings, related to Figure 2 and Figure S5

Timeline	Operation	Cell density ($x10^6$/mL)
Day 0	seeding HEK293T	0.3
Day 1	transfection	0.6
Day 2	24-h post transfection	1.2
Day 3	48-h post transfection	2.4
Day 3	dilution prior to patch clamp	0.48

Table S5: Ankyrin repeats in monomeric structure models of TRPAs, related to Figure 3

Channel	*Rhodnius prolixus*	*Drosophila melanogaster*
TRPA1	17	17
Painless	10	10
Waterwitch	9	10
Pyrexia	-	9
TRPA5B	14	-

Table S6: Comparison of residues in the upper and lower gate between the AlphaFold tetrameric model and determined structures of dTRPA1, related to Figure S9

Alignment	7YKS (3.0 Å) RMSD Cα (Å)	7YKS RMSD rejected outliers (Å)	7YKS RMSD (Å)	7YKR (3.2 Å) RMSD Cα (Å)	7YKR RMSD rejected outliers (Å)	7YKR RMSD (Å)
Tetrameric model	0.328	0.335 (120/144 atoms)	0.686	0.516	0.478 (119/144 atoms)	0.737
7YKR (3.2 Å)	0.352	0.352 (124/144 atoms)	0.674			

Table S7: Oligonucleotide primer sequences, related to Figure 2, S3, S5.

ORF primers	Sequence (5' - 3')	Restriction site	Amplicon size (bp)
s,forward; as, reverse			
rTRPV1-s*	*cggcc*gcgatcgc**GCCACC**ATG**G**AACAACGGGCTAGCTTAGAC	AsiSi	2,550
rTRPV1-as	*ggtgcc*acgcgtTTTCTCCCCTGGGACCATGGAATC	MluI	
dTRPA1D-s*	*cggccg cttaag* **GCCACC**ATGCCCAAGCTCTACAACGGAGT	AflIII	3,696
dTRPA1D-as	ggtgcccgtacgCATGCTCTTATTGAAGCTCAGGGCGG	BsiWi	
Rp-TRPA5B-s	*cggcc*gcttaag**GCCACC**ATG**G**ACAGTCTAGTGCCTGCGATACAG	AflII	3,456
Rp-TRPA5B-as	*ggtgcc*cgtacgAACATAATTAGGATGTGTTGCGCC	BsiWi	
**pcDNA5 construct verification primers**			
pcDNA5-s	GCTGTTTTGACCTCCATAGAAGA	-	-
pcDNA5-as	TAGAAGGCACAGTCGAGG	-	-
pLenti-s	ACCGCATGTTAGCAGACTT	-	-
mRuby-as	CGGCGGCTTAAACCTTATCGTCG	-	-
**qPCR primers**			
Actin-s	ACATCAGGAAGGACTTGTACGC	-	113
Actin-as	TTTGATGGTTGATGGAGCCAGG	-	
Rp-TRPA5B-s	GATGTTACTGGACTTCATGCGC	-	86
Rp-TRPA5B-as	TATTTTTCTTGTTCGTTTGTCCTTTACTTCT	-	

underlined sequence: restriction sites (RS); sequence in italics: extra bases; sequence in bold: Kozak

* Information regarding source plasmids:

rTRPV1 clone = rat TRPV1 pFBF1 (pCDNA3-CFLAG-RnTRPV1-N604S) (Provided by Rachel Gaudet)

dTRPA1-D = Fruit fly "TrpA1-PG type=CDS (A10a) =TRPA isoform "D" (Provided by Pengyu Gu)

Data S1: List of all source data, related to Figure 1,2,3, and Figures S1 to S10.

Figure 1 uses *Rhodnius_RNAseq.xlsx*

Source data file : https://figshare.com/s/2d1ad932f34bdca2a769

Figure 2 uses *Negative cells.zip, rTRPV1.zip, dTRPA1.zip, RpTRPA5B.zip, Source_data_analysis_Fig2_FigS5.xlsx, Fig2D_FigS5_rTRPV1.xlsx, Fig2F_FigS5_dTRPA1D.xlsx, Fig2G-2L_RpTRPA5B.xlsx, Rp_Data Final analyses.pzfx*

Source data files : https://figshare.com/s/995be46d32030cd52910

Figure 3A uses *dTRPA1_tetrameric_model….pdb*

Source data file :https://figshare.com/s/0606a76fe5e96202ef73

Figure 3B uses *Monomeric_TRP_models.pse,* and pdb files:
dPain_A_43df7_relaxed_rank_1_model_3
dPyx_A_fd40c_relaxed_rank_1_model_3, dTRPA1_A_80135_relaxed_rank_1_model_4
dWtrw_A_a1a85_relaxed_rank_1_model_3, RpPAIN_A_9bf5c_relaxed_rank_1_model_3
RpTRPA1isoformA_exon14A_A_bc969_relaxed_rank_1_model_4,
RpTRPA5B_A_b3925_relaxed_rank_1_model_3, RpWTRW_A_43565_relaxed_rank_1_model_3

Source data files :https://figshare.com/s/0606a76fe5e96202ef73

Figure 3C-G use *RpTRPA5B_tetramer.pdb*, *RpTRPA5B_tetramer.pse*

Source data file :https://figshare.com/s/0606a76fe5e96202ef73

Figure S1 uses *FigS1_Insect_TRPA.fasta*, *FigS1_Insect_TRPA.nwk*, *FigS1_Insect_TRPA.Rmd FigS1_TRPAtree.pdf (High quality file)*

Source data files https://figshare.com/s/aca16873b4cc7e62d242

Figure S2 uses *FigS2_Insect_TRPA5.fasta*, *FigS2_Insect_TRPA5.nwk*, *FigS2_Insect_TRPA5.Rmd*

Source data files: https://figshare.com/s/014ec6a509c3c3d01896

Figure S3 uses *qPCR.csv*, *Rhodnius_qPCR.Rmd*

Source data files https://figshare.com/s/09aa5dfd07dc56ee1bcc

Figure S4 uses *A_membrane.tif, A_Exp2sec.tif, B_membrane.tif, B_Exp10sec.tif, C_membrane.tif, C_Exp5.8sec.tif*

Source data files: https://figshare.com/s/85aa2e7bfcd7a3bc4168

Figure S5 uses *rTRPV1.zip, dTRPA1.zip, Source_data_analysis_Fig2_FigS5.xlsx, Fig2D_FigS5_rTRPV1.xlsx, Fig2F_FigS5_dTRPA1D.xlsx, Rp_Data Final analyses.pzfx*

Source data files: https://figshare.com/s/995be46d32030cd52910

Figure S6 uses *open pipette kinetics.pzfx*

Source data file: https://figshare.com/s/789896f4ab3b1804a648

Figure S7 uses *dPain_A_43df7_relaxed_rank_1_model_3*, *dPyx_A_fd40c_relaxed_rank_1_model_3*, *dTRPA1_A_80135_relaxed_rank_1_model_4*, *dWtrw_A_a1a85_relaxed_rank_1_model_3*, *RpPAIN_A_9bf5c_relaxed_rank_1_model_3*

RpTRPA1isoformA_exon14A_A_bc969_relaxed_rank_1_model_4, RpTRPA5B_A_b3925_relaxed_rank_1_model_3, RpWTRW_A_43565_relaxed_rank_1_model_3

Source data files :https://figshare.com/s/0606a76fe5e96202ef73

Figure S8 uses *dTRPA1_tetrameric_model….pdb*

*Source data file :*https://figshare.com/s/0606a76fe5e96202ef73

Figure S9 uses *RpTRPA5B_tetramer.pdb*, *RpTRPA5B_tetramer.pse*

*Source data files :*https://figshare.com/s/0606a76fe5e96202ef73

Figure S10 uses *RpTRPA5B_tetramer.pdb*, *RpTRPA5B_tetramer.pse*

*Source data files :*https://figshare.com/s/0606a76fe5e96202ef73

References

[S1] Liu B, Hui K, Qin F. 2003. Thermodynamics of Heat Activation of Single Capsaicin Ion Channels VR1 *Biophys J.* **85**: 2988–3006. (10.1016/S0006-3495(03)74719-5).

[S2] Yao J, Liu B, Qin F. 2010. Kinetic and energetic analysis of thermally activated TRPV1 channels. *Biophys J.* **99**, 1743-1753. (10.1016/j.bpj.2010.07.022).

[S3] Caterina MJ, Schumacher MA, Tominaga M, Rosen TA, Levine JD, Julius D. 1997. The capsaicin receptor: a heat-activated ion channel in the pain pathway. Nature **389**: 816-824 (10.1038/39807).

[S4] Tominaga M, Caterina MJ, Malmberg AB, Rosen TA, Gilbert H, Skinner K, Raumann BE, Basbaum, AI, Julius D. 1998. Neuron **21**: 531-543 (10.1016/s0896-6273(00)80564-4).
